# Comprehensive Needs Assessment for Workplace Health Promotion: A Case Study of the University of Bern

**DOI:** 10.3389/ijph.2025.1608274

**Published:** 2025-09-18

**Authors:** Carolin Glauser, Ivana Igic, Thomas Berger, Alex Bertrams, Achim Elfering, Barbara Engel, Tina Hascher, Jennifer Inauen, Miriam Lüthi, Claudio R. Nigg

**Affiliations:** ^1^ Institute of Sport Science, University of Bern, Bern, Switzerland; ^2^ Institute of Psychology, University of Bern, Bern, Switzerland; ^3^ Military Academy ETH Zürich, Zürich, Switzerland; ^4^ Institute of Educational Science, University of Bern, Bern, Switzerland; ^5^ Personnel Department, University of Bern, Bern, Switzerland

**Keywords:** health, employee, worksite, needs assessment, promotion, university

## Abstract

**Objectives:**

To understand the holistic state of work conditions, health and wellbeing of University of Bern employees through a social-ecological lens.

**Methods:**

A comprehensive assessment included analysis of existing measures, stakeholder mapping, employee survey, expert interviews, a reflection and evaluation instrument, policy analysis, and environmental analysis.

**Results:**

Integrating results revealed that the University provides numerous employee health opportunities such as broad variety and frequency of university sports options, good working climate, ergonomic workplace, well-developed and centrally established communications department, extensive range of Bernese universities independent counselling center services, and some relevant health promotion policies. However, these opportunities are not integrated into a systematic health strategy. Further analyses indicated four fields of action: 1) to anchor health promotion in the university culture and organization; 2) to promote mental health, wellbeing, and stress management; 3) to improve health promotion communication; and 4) to ensure sustainability, to implement continuous process and outcome evaluation of the actions.

**Conclusion:**

This could serve as a blueprint model for universities ensuring a holistic understanding of health and wellbeing, and informing related practical implications and organizational health governance.

## Introduction

Recent globalization and technologization have profoundly transformed work, altering working conditions and increasing stress in industrialized countries [1]. Prolonged exposure to demanding psychosocial working conditions heightens risks to mental and physical health [1, 2]. Excessive work stress might lead to harmful coping behaviors like overuse of nicotine, alcohol, or medications, and neglect of recreation [3]. It also increases the likelihood for burnout [3] or depression [4], and non-communicable diseases like cardiovascular conditions [5] or musculoskeletal disorders [6]. Chronic workplace stress also impairs cognitive performance, reducing productivity, and increasing absenteeism and turnover [7–13].

The past 2 decades have reshaped university working conditions [14]. The 1990s saw universities characterized by autonomy and low stress, but recent years have brought an unhealthier environment [14, 15]. Increased professional demands on university employees have led to unhealthy coping behaviors and stress-related physical complaints [16–19]. Thus, strategic health promotion and prevention at universities are crucial.

Health promotion is transdisciplinary [20], evidence based, and rooted in the Ottawa Charter [21]. The Charter emphasizes a holistic view of health, integrating physical, mental, and social wellbeing, and encourages empowerment and reduction of health inequalities [22]. Workplace health promotion includes joint measures by employers, employees, and society to improve workplace health [23, 24]. Effective health promotion reduces stress-related illness costs, absenteeism, turnover, and increases productivity and attractiveness of companies [25]. It positively impacts mental health, substance use, physical activity, diet, weight, and presenteeism [26].

Universities with high health awareness are effective environments for health promotion, as recognized by the WHO’s “Health Promoting Universities” project [27, 28]. Despite challenges like institutional complexity and diverse target groups [29], universities can offer significant potential for holistic health promotion (addressing multiple dimensions of health—physical, mental, emotional, social, and spiritual; [30–32]) and implement systematic, multi-component approaches that prove to be more successful than isolated efforts [28, 33–35].

**FIGURE 1:**
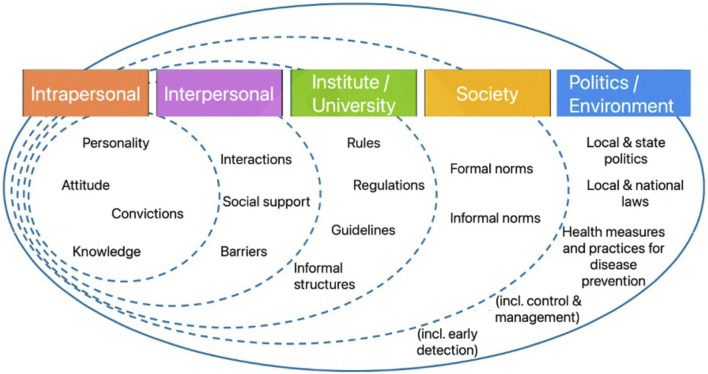
Social-ecological model for universities (42; Gesunde UniBE, Switzerland, 2021).

A suitable approach for universities is a so-called “setting approach”, addressing health where people spend most of their time [36, 37]. This approach, central to the Ottawa Charter, has underpinned public health promotion since the 1980s [38], integrates behavioral and structural prevention and utilizes a resource-oriented method, capitalizing on existing communication and action patterns [39–42]. Understanding universities as complex systems involves recognizing them as dynamic ecosystems that encompass interactions between “organisms” and their environment [42, 43]. The social-ecological framework helps comprehend these interactions, incorporating multiple levels from individual to policy, and addressing the complexity of human conditions [44–47]. Figure 1 illustrates this model as applied to universities, based on Bronfenbrenner’s ecological framework [48].

The intrapersonal level focuses on improving factors within an individual. For example, Johnston et al. [49] used individualized in-person screening to identify current health status and predict a participant’s future disease risk and prognosis along with follow-up health coaching mainly conducted directly via e-mail among university staff. This illustrates interventions targeting the individual’s attitudes and their motivation within a university setting.

At the interpersonal level, interactions between people are addressed. In the study by Breaux-Shrosphire et al. [50], employees participated in a 10-week team-based weight loss competition, demonstrating the impact of group dynamics on health behaviors.

The institution or university level encompasses organizational structures such as physical environment, rules, or regulations specific for the University to support health. A good example of institutional level efforts affecting entire worksites for health promotion (outside the University context) is a study conducted on predominantly manufacturing work sites reported on the effectiveness of an intervention including: 1) joint worker-management participation in program planning and implementation, operationalized through an employee advisory board and a designated work-site liaison; and 2) consultation by project staff with management on worksite environmental changes, including tobacco control policies, increased availability of healthy foods, and reduction in the potential for exposure to occupational hazards [51].

The societal level includes broader social norms and values. For instance, the “Healthy Campus Bonn” project [52] enhanced student health literacy and positioned the university as a key player in shaping societal health norms. This level highlights the university’s role in extending health influences beyond its immediate community.

At the policy level the focus shifts to engaging with policymakers and implementing broad-scale initiatives. This includes presenting research findings that inform public health policies and organizing health campaigns, such as the coronavirus vaccination efforts in Western countries [53]. Such activities demonstrate the university’s capacity to influence public health policy and practice.

The Centers for Disease Control and Prevention [54] recommend simultaneous interventions at multiple levels to counteract health-damaging behavior. This multi-level approach, exemplified in the university setting, underscores the potential for comprehensive strategies that engage individuals, social groups, institutions, and policymakers in promoting public health.

Despite their scientific orientation, universities often seem to fail short in integrating research findings into their environment to enhance workplace health [55, 56]. Thus, a needs assessment is recommended to establish a baseline, identify ongoing efforts, and prioritize health promotion measures [57, 58]. However, there is a lack of documented comprehensive university health needs assessments in the literature, indicating a gap in shared knowledge and best practices. This paper aims to address this deficiency by presenting a comprehensive university employee health needs assessment conducted through the social-ecological framework within the “Healthy University Bern” initiative. This will not only offer insights into current health challenges but also set a precedent for future health interventions in university settings.

## Methods

For the comprehensive and systematic assessment of the current state of employee health promotion and prevention at the University of Bern, as well as the associated planning and prioritization of improvement potentials, a needs assessment was developed and conducted. This assessment encompassed seven approaches addressing purposes through different social-ecological levels (see Table 1). The methodological design adhered to the initial seven quality criteria outlined by the Working Group on Health-Promoting Universities [59]. German or English was used, per participant preference.

**TABLE 1:** Methods of the University of Bern needs assessment by Social-Ecological Model levels (Gesunde UniBE, Switzerland, 2021).

Needs Assessment method	Data collection year: 2021	Intrapersonal	Interpersonal	Institute/University	Society	Politics/Environment
Analysis of existing measures	17.05. – 08.09.	X	X	X		
Stakeholder Mapping	16.02. – 13.08.		X	X	X	
Employee Survey	03.11. – 22.11.	X	X	X		X
Expert Interviews	21.10. – 13.12.		X	X	X	X
REI-Structure	12.10. – 13.12.			X	X	X
Policy Analysis	27.07. – 18.11.					X
Environmental Analysis	13.10. – 08.11.					X

The University of Bern is a comprehensive public research university founded in 1834, is self-governed, regulated and financed by the Canton of Bern, and offers a broad choice of courses and programs in eight faculties and some 150 institutes. The University of Bern is spread over 18 sites in the city of Bern.

### Analysis of Existing Measures

Sample: To gain an overview of existing workplace health promotion measures at the University of Bern, various sources were consulted, including the university website, the most recent (2018) employee satisfaction survey, the Human Resources Office, the faculties, and health institutions within the City and Canton of Bern.

Data analysis: These health promotion measures were first identified and then categorized in topic areas.

### Stakeholder Mapping

Sample: A stakeholder analysis [60] was conducted to identify influential stakeholders affecting the success of health promotion efforts among university employees.

Data analysis: Members of the personell department and the Healthy UniBE commission identified internal and external stakeholders and separately categorized them based on their interest, influence, power, and attitude toward health promotion using a scale (low, medium, high) evaluated through consensus by the healthy UniBE commission.

### Employee Survey

**FIGURE 2:**
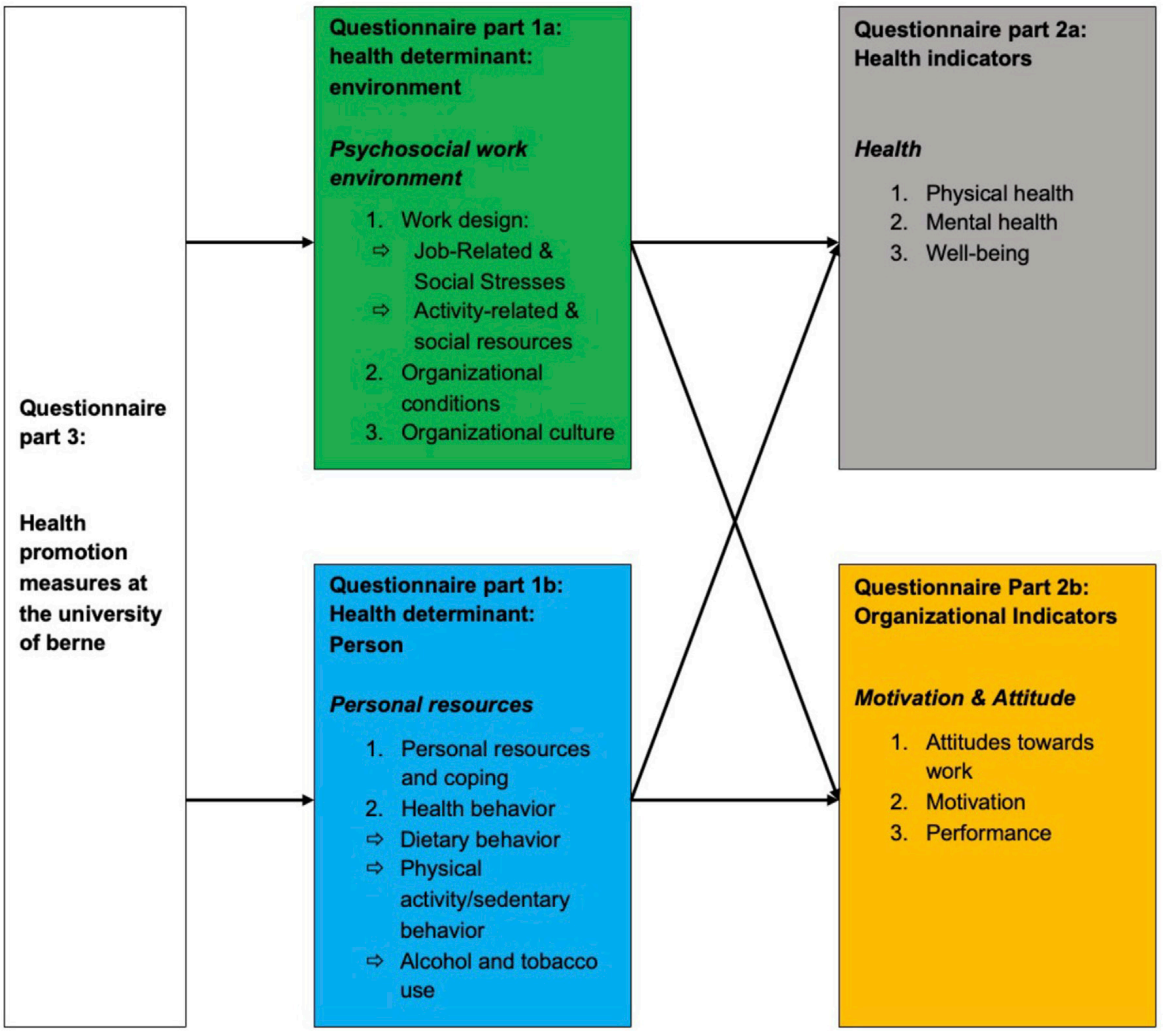
University employee survey content and structure (Gesunde UniBE, Switzerland, 2021).

Sample: The employee survey (for content/structure see Figure 2) used predominantly validated questions based on the Job Demand Control Model [61], the Job Demand-Resource Model [62], job stress index [63] and various university-specific instruments, was piloted at the Faculty of Human Sciences from 03.11.2021 to 22.11.2021. After data cleaning, 36% (N = 182/507) of the employees were included in the analyses.

Data analysis: The survey gathered insights on employees’ perceptions of existing measures, job-related stressors, resources, organizational conditions, personal resources, health behaviors, and demographic factors. Selected variables are shown in Supplementary Tables S2–S5.

### Expert Interviews

Sample: Interviews regarding seven health promotion related topics (knowledge of health promotion, potential for improvement, factors for long-term success, structure, focus topics, wishes, and demands such as collaboration opportunities) were conducted with key individuals (in writing n = 12; or orally n = 8; participation rate 20/26 = 77%) between 21.10.2021 and 01.11.2021.

Data analysis: Qualitative content analysis [64] of interview responses resulted in nine categories and six subcategories representing various aspects of university health promotion. Three interviews were coded independently by two raters resulting in good inter-rater reliability (Cohen`s kappa coefficient = 70%) [65].

### Reflection and Evaluation Instrument

Sample: A total of 15 relevant stakeholders at the operational level (representing Management, University Sports, Human Resources, the Departments of Communication, Gender Equality, Quality, Risk Management, and the mid-level faculty) provided feedback via a reflection and evaluation instrument (REI; [66]) either at a workshop or via email. The REI focuses strengths and development potentials of workplace health promotion addressing four topics: organisational structures and processes, existing analyses, internal and external communication, and health promotion and prevention measures.

Data analysis: Assessment sheets were analyzed based on operationalized quality criteria (which are 165 themes (assessed by 2-8 items, reflecting each criteria) focusing on organizational structures (14 themes), communication (6 themes), and health promotion measures (6 themes).

### Policy Analysis

Sample: Thirty-six internal documents (see Supplementary Table S1) were analyzed to evaluate policies related to health promotion at the University of Bern.

Data analysis: First, internal documents were reviewed that relate to the promotion and prevention of health, with a focus on the internal policies of the university. Various offices at the University of Bern were consulted, including: the Coordination Office for Sustainable Development, the Coordination Office for Early Career Researchers, the University’s Legal Services, the Office for Gender Equality, the Quality Assurance Office, and the leadership of the Faculty of Humanities. Second, keywords were used to search within the documents.

The identified policies were read and categorized into two groups: those that directly address the promotion and prevention of employee health and wellbeing, and those that indirectly relate to the promotion of personal and environmental health determinants according to a thematic content analysis.

### Environmental Analysis

Sample: The analysis was conducted at 12 university sites using a developed university-specific checklist focusing on physical activity, nutrition, relaxation, smoking, passive smoking, and health-promoting information environments (primarily based on [67–69]).

Data analysis: Data from site inspections were analyzed to assess health-promoting aspects across different locations. Two university sites were analyzed by three independent observers each (resulting intraclass correlation coefficient r = 0.96/.91).

## Results

Summaries of results from the seven needs assessment approaches are presented below.

### Analysis of Existing Measures

Supplementary Figure S1 shows the 11 overarching health areas in which the University of Bern is engaged. The university includes health promotion in some key regulations and provides action recommendations. There are existing offers for physical activity (Uni Sport) and a discrimination-free, respectful work and leadership culture. Additionally, the University of Bern provides favorable conditions for promoting health (such as sports programs, bicycle-friendliness, ergonomic workspaces, healthy food options, and available break rooms). Personal resources (like self-efficacy), health behaviors (such as exercise, sport, nutrition, smoking, and alcohol consumption), wellbeing, life satisfaction, physical health, job satisfaction, emotional attachment to the University of Bern, and work engagement were also rated positively.

### Stakeholder Mapping

In sum, 13 internal and 12 external stakeholders were identified and ranked on a scale of one to five based on their interest, influence, power, and attitude towards university employee health (Supplementary Figure S2).

The majority of stakeholders (23 individuals) displayed a neutral to positive attitude towards the project, with a medium to high level of interest. Their influence ranged from very low to very high (scale 1–5). Only two stakeholders exhibited a somewhat negative attitude towards the project, with very low interest (interest = 1) but medium influence (influence = 3).

Nine internal stakeholders demonstrated very high interest (Interest = 5), with high influence (Power/Influence = 3–4) and a positive attitude (Attitude = 5). External stakeholders (8 individuals) generally showed significant interest (Interest = 4), albeit with low to moderate influence (Influence = 1–2) and a slightly positive to positive attitude.

### Employee Survey

**FIGURE 3:**
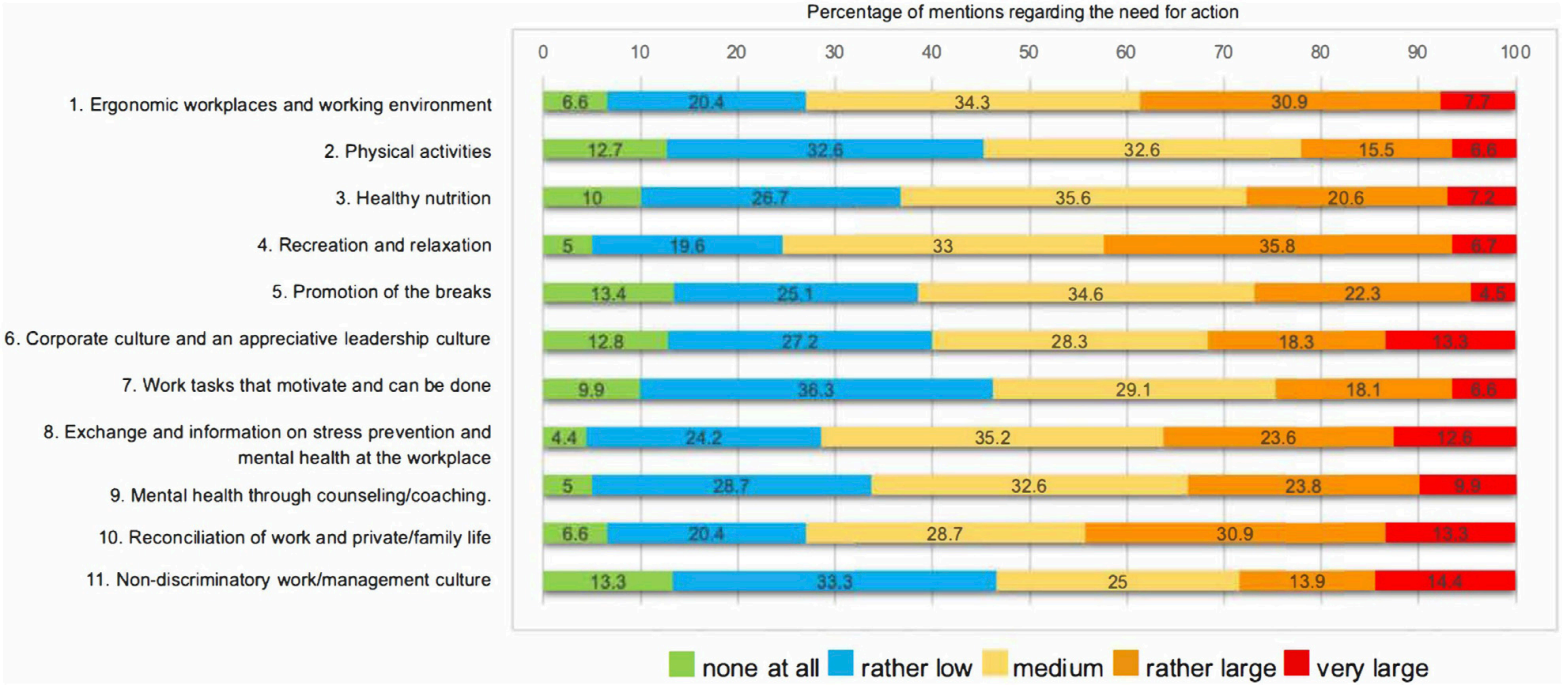
Estimation in percent of the need for action across eleven different health areas from the university employee survey (Gesunde UniBE, Switzerland, 2021).

Figure 3 displays the percentage of self‐reported mentions regarding the need for action across the 11 different health areas from the first part of the employee survey.

Supplementary Tables S2–S5 show the need for improvement across four main survey categories (environment, personal, health and organizational indicators), based on comparison samples or guidelines, using color coding: green indicates that the University’s results exceed guidelines, orange signifies compliance with guidelines, and red indicates results below guidelines. White cells indicate where no representative comparison samples or guidelines were available. Significant differences (p < 0.05) related to gender, employment relationship, and functional differences are denoted with “*.” Additional details on significance tests (p < 0.05) concerning gender, employment relationship, and functional differences can be found in Supplementary Table S6. Due to the exploratory nature *p* was set at 0.05.

Most of the areas sampled (13 in total) showed similar or superior results compared to representative comparison samples and health guidelines. However, results for seven variables (Qualitative Overload, Job Insecurity, Sitting Behavior, Work Related Emotional Exhaustion, General Job Satisfaction, Work Engagement, Presentism) indicate a need for improvement compared to these benchmarks. To reflect the data best, pairwise deletion was used for missing data.

### Expert Interviews

Supplementary Table S7 presents the topics discussed by at least three interviewees per interview block. Sample Quotes (original German quotes in Supplementary Table S8) support three strengths regarding health promotion at the University of Bern. Firstly, university sports was highlighted for its diverse offerings and varied sports programs available to employees. Briefly, university sports offer a variety of inclusive, noncompetitive physical activities for students and staff, including regular courses, training sessions, and occasional events. While some competitive options are available, the focus is on accessibility for all skill levels. Secondly, the ergonomic workplace, particularly the height-adjustable desks, was identified as a strength. Thirdly, numerous collaborations were noted between the “Healthy University of Bern” initiative and vice rectorates, deaneries, and other departments.

The interviews also identified significant areas for improvement including stress management and prevention, as well as the promotion of mental health (n = 14), reducing workload to improve work-life balance (n = 13), fostering a sustainable health-promoting organizational culture (n = 9) and establishing a central coordinating office or position for university health promotion (n = 13).

### Reflection and Evaluation Instrument

REI strengths and areas for development are presented concerning organizational structures and processes, existing analyses, internal and external communication, as well as health promotion and prevention measures at the University of Bern. High priority potentials highlighted in grey (with low to moderate effort and moderate to high benefits) in Supplementary Table S9 are identified for each REI area.

#### Organizational Structures and Processes

Strengths identified include the integration of health-promoting aspects into staff regulations, structural incorporation of health promotion within university management, establishment of a steering committee with regular meetings, initiation of networking activities focusing on health promotion, goal definition and monitoring across equality, quality, and sustainability areas, and a predominantly exercise-friendly infrastructure.

#### Existing Analyses

The University conducts a representative employee survey every 4 years through an external agency (Empiricon AG) addressing various health sub-areas. Additionally, a holistic questionnaire developed during the needs analysis provides evidence-based insights into health.

#### Internal and External Communication

Strengths include a well-established and centrally located communications department and the identification of target groups through the needs analysis.

#### Health Promotion and Prevention Measures

Strengths identified include the comprehensive services offered by the independent counseling center of Bernese universities, collaboration between this counseling center and the University of Bern’s Human Resources department, target-specific training and ongoing education programs, and a diverse range of services provided by the university sports center.

In summary, key development potentials in health promotion include defining targets in the health cross-sectional area, integrating “health” into the University of Bern’s strategy and mission statement with involvement from university management, and expanding health-promoting initiatives at the behavioral level.

### Policy Analysis

Of the 36 documents reviewed, nine policies directly related to the promotion and prevention of health and employee wellbeing. The remaining 27 policies have an indirect relationship with health promotion and prevention and employee wellbeing. Supplementary Table S10 shows the number of documents and policies identified in various health-related topics (cf. [70]). The University of Bern has a range of policies focused on promoting and preventing health emphasizing mental and physical health, safety, personal integrity, and the sustainable management of social resources.

### Environmental Analysis

Supplementary Table S11 shows the health-promoting infrastructures available at the 12 sites and their immediate surroundings. For each site, a point score was calculated for the domains of physical activity, exercise, and sport; nutrition; relaxation and recreation; smoking and second-hand smoke; and the information environment.

#### Physical Activity, Exercise, and Sports

All locations offer physical activity opportunities within a five-minute walk. All sites have bicycle parking with seven sites having adequate number of bike racks; four sites mediocre, and one site inadequate. Ten of the twelve sites have bicycles available for rent within 200 m. Showers are available at ten sites, and locker rooms are available at eight sites. Only two sites have fitness rooms.

#### Nutrition

All sites analyzed have at least one dining room. On a scale of 0–3, employees rated the facilities of the dining rooms as high on average (M = 2.35, SD = 0.46). The condition of the dining canteens was also rated as high, with a mean of 2.73 (SD = 0.46) on a scale of 0–3. There are a total of ten dining halls or cafeterias at eight of the twelve sites. Seven dining halls or cafeterias identify allergenic ingredients, and four of these label the nutritional values (Nutri-Score) of the food. Tap water is safe in Bern and six locations provide water refill facilities apart from those in restrooms. Ten sites have food or beverage vending machines, but only 31.3% (SD = 6.6%) of the compartments contain healthy foods.

#### Relaxation and Recreation

All sites have infrastructure for breaks or rest/relaxation opportunities. Three sites have explicit rest/relaxation rooms or dormitories. The majority of sites (N = 10) are rated as having some decorative appeal and a pleasant atmosphere. Eleven sites have infrastructures that invite outdoor breaks. Four sites each feature at least table tennis or table soccer.

#### Smoking and Passive Smoking

Only two sites have a partial or complete smoking ban in the outdoor areas. At all other sites (N = 10), ashtrays mostly invite people to smoke directly in front of the entrance doors of the buildings.

#### Information About Health-Promoting Offers

All university sites analyzed lack signs, posters, prompts, etc., regarding health topics or health behaviors. On average, there is a notice on one in ten floors regarding nutrition, health promotion, or advertising employee support programs (M = 0.10 - 0.12, SD = 0.12 - 0.25). Most of the print media are posters from university sports. The health topics of smoking and alcohol are non-existent at all sites analyzed.

### Integrated Analysis and Resulting Action Foci

The results of the seven forms of data collection used in the needs analysis can be summarized into four fields of action.

First, the University of Bern lacks a uniform definition of health, as reflected in the policy analysis. The need to include health promotion in the university’s mission statement is evident from both the policy analysis and the REI. The expert interviews and the REI reveal a desire for a central coordinating body. These findings identify the first field of action: the integration of health promotion and prevention into the university’s culture and organization.

Second, the employee survey and expert interviews highlight the need for measures in mental health, wellbeing, stress management, work-life balance, and recovery. The environmental analysis underscores the urgent need for improvements in the ambience and equipment of break rooms. Together, these results point to the second field of action: promoting mental health and wellbeing and stress management.

Third, the employee surveys and expert interviews indicate that existing health promotion measures need better publicity. The environmental analysis strongly suggests that smoking regulations and the posting of decision prompts and posters are urgently needed. These findings outline the third field of action: improving communication regarding health promotion measures.

To ensure the sustainable implementation of these fields of action, a continuous process and result evaluation with built-in feedback loops is essential. Therefore, the fourth field of action is: evaluation and quality.

Considering priority, feasibility, likelihood of short-term impact, university readiness, resources, and strategy, the University of Bern will initially focus on the second field of action—promoting mental health and wellbeing and stress management.

## Discussion

This paper presents the methods and results of a comprehensive university health needs assessment conducted within the “Healthy University Bern” initiative, viewed through the social ecological lens. The assessment consisted of seven components: analysis of existing measures, stakeholder mapping, employee survey, expert interviews, reflection and evaluation instrument (REI), policy analysis, and environmental analysis. These components reflect the holistic nature of the assessment and the different levels of the social ecological model.

Integrating the results from these components revealed the following:

a) The University of Bern offers numerous opportunities for employee health and wellness, including varied university sports, a good working climate, ergonomic workplaces, a well-developed communications department, extensive services from the independent counseling center for Bernese universities, and various policies promoting and preventing health.
b) Four areas of future action can be identified:
  1. Anchoring health promotion and prevention in the university culture and organization.
  2. Promoting mental health and wellbeing and stress management.
  3. Improving communication regarding health promotion measures.
  4. Evaluation and quality assurance.

In terms of anchoring health promotion and prevention in the university culture and organization, several recommendations are proposed (the intended level of the social ecological model provided):

• Developing and implementing a concept for health and health promotion, ideally with the establishment of a central coordination office, a practice shown to be beneficial across several universities ([71]; University).
• Standardizing the definition of health and health promotion across all university documents. Proposed definitions emphasize health as a holistic concept encompassing physical, mental, and social wellbeing, while health promotion is viewed as empowering individuals to take control of their health and influence key determinants ([72], Policy).
• Establishing a comprehensive university health policy (Policy).
• Integrating health promotion and prevention measures closely with other university areas, such as quality assurance, diversity promotion, sustainable development in teaching, research, and administration, human resources, risk management, career services, and the coordination office for the promotion of young scientists. Universities adhering to Health Promoting Universities criteria collaborate across these services to enhance health promotion efforts ([73], University).
• Expanding the “Healthy University of Bern” initiative to involve additional faculties and interested internal and external stakeholders, a strategy proven beneficial [73, 74], with active engagement from all stakeholders being crucial ([74], Society).
• Involving students actively in the initiative (University).
• Establishing and networking health structures within faculties and competence centers, providing essential support for university health promotion ([71], University).
• Adapting and extending the employee survey used in other faculties at the University of Bern.
• Integrating the employee survey with the existing personnel questionnaire tool (University).
• Defining expanded action areas for each faculty, with ongoing goal-setting aligned with health promotion objectives as per the criteria of the Okanagan Charter ([71] - the international guide for health promoting Universities and Colleges, University).
• Networking the University of Bern with other universities and networks, fostering cooperation to share insights and best practices ([74], Society).
• Increasingly embedding the initiative within university structures (University).
• Clarifying human and financial resource requirements at the University of Bern, prioritizing these resources as critical for successful implementation of health promoting strategies according to the Okanagan Charter criteria ([71, 73], University).

Recommendations for promoting mental health, enhancing wellbeing, and stress management include:

• Forming interdisciplinary working groups across faculties to conceive, plan, and evaluate health promotion measures (Interpersonal).
• Expanding programs aimed at improving mental health and wellbeing, particularly in reducing stress sources, both at the organizational and individual levels (Intrapersonal, University).
• Facilitating work-life balance through flexible scheduling and spatial arrangements (Environment).
• Developing tailored programs for junior researchers and employees on fixed-term contracts to enhance mental health, wellbeing, and stress management (Intrapersonal, Interpersonal).

Recommendations for improving communication about health promotion measures include:

• Incorporating health promotion and prevention topics into the communication channels of the University of Bern’s human resources and promotion policies. Effective communication strategies and structures, as noted by Squires and London [71], support internal collaboration on health promotion (University, Policy).
• Ensuring visibility of existing health-promoting initiatives and services to all University of Bern employees, with simplified access to information ([71], Interpersonal, Society).
• Centralizing communication of new initiatives and measures across the university (University).

These actions contribute to enhancing the University of Bern’s reputation as a health-promoting “Healthy University” externally.

Recommendations for evaluation and quality assurance include:

• Developing logic models and evaluation plans for each action area.
• Conducting regular process evaluations with built-in feedback mechanisms [73, 75].
• Performing outcome evaluations biennially, utilizing self-evaluation, benchmarking against other universities, or external evaluations [76].
• Pursuing external accreditation as a quality criterion for university health promotion and prevention efforts, thereby enhancing the initiative’s visibility.

A significant strength of the needs assessment lies in its comprehensive approach to employee health and wellbeing. Beyond addressing working conditions, organizational factors, and environmental influences, it also examines employees’ mental health. By employing seven distinct components across various levels, the assessment provides at least two methods for each tier of the social ecological model (intrapersonal, interpersonal, institution/university, societal, political/environmental). This approach ensures a rich diversity of information sources spanning all categories of the social ecological model.

For instance, while a questionnaire offers insights into the perceived environment, environmental observation provides a more objective characterization that complements these perceptions. For example, the employee survey revealed that approximately 5% of Faculty of Human Sciences employees smoke regularly. In contrast, environmental analysis indicated that only two locations enforce partial or complete smoking bans outdoors, while ashtrays at the remaining ten sites often encourage smoking near building entrances. Similarly, while a Reflective and Evaluative Instrument (REI) captures perceived environmental aspects, a policy analysis provides an objective assessment that supports these findings. Both the REI and policy analysis underscored the necessity of integrating health promotion into the university’s mission statement.

The use of a multi-component approach and the resultant diverse information sources across all socio-ecological model categories (cf. [48]) distinguishes the University of Bern’s needs assessment from those of other universities. For instance, the University of Limerick [77] focused its action areas solely on extensive surveys of staff and students, policy analyses, and stakeholder inputs, thus not fully exploiting complementary information sources as per the socio-ecological model (cf. [48]). Conversely, the University of Bielefeld has implemented numerous health-promoting initiatives derived from a comprehensive concept with seven core components [78, 79], the Bielefelder questionnaire [80], and health circles [81]. However, this approach may result in less information density compared to the comprehensive needs assessment of the University of Bern. Similarly, the University of Bonn has extensive health-promoting measures primarily informed by stakeholder analyses, online surveys, and program evaluations [52], lacking the depth of a detailed needs assessment. Meanwhile, the University of Lancashire’s Self-Review Tool (SRT) offers insights into a university’s health status under the Healthy University approach [74, 82], yet it serves more as a guiding framework than a detailed needs assessment.

The decision to focus on promoting mental health and wellbeing and stress management in the University setting aligns well with the socio-ecological model by addressing multiple levels of influence on health [42]. At the core, the initiative empowers individuals by promoting self-awareness and personal strategies for mental wellbeing. For example, focusing on adding a mental-health focus to the University’s App would enable personalized interventions. At the interpersonal level “Gesunde UniBE” can foster supportive relationships among peers, faculty, and staff. By encouraging open dialogues about mental health and providing resources for stress management, the initiative strengthens social support networks, which are crucial for mental wellbeing. The initiative should integrate mental health promotion at the organizational level into the university’s policies and practices. This includes training programs for staff, creating supportive environments, and embedding wellbeing into the organizational culture, thus facilitating systemic change. By collaborating with local organizations and health services, “Gesunde UniBE” would extend its impact beyond the university. These partnerships enhance community engagement and ensure that mental health resources are accessible to a broader population. The initiative would contribute to societal change by participating in research and policy development aimed at improving mental health at a national level. By sharing findings and best practices, “Gesunde UniBE” influences broader health promotion strategies. The “Gesunde UniBE” initiative exemplifies a comprehensive approach to mental health promotion, effectively operationalizing the socio-ecological model through interventions at individual, interpersonal, organizational, community, and societal levels.

Conclusively, the University of Bern’s comprehensive needs assessment could serve as a blueprint for other universities aiming to conduct thorough evaluations, ensuring a holistic understanding of health and wellbeing among their stakeholders.

### Limitations

A limitation of the needs analysis is its timing in 2021, during which workplace conditions were affected by the COVID-19 pandemic, resulting in a non-standard work environment (with increased remote work), despite the University of Bern maintaining operations. Tancredi et al. [83] demonstrated that depression, anxiety, and stress levels in a representative Swiss sample were significantly higher than usual. In the context of the needs analysis, this suggests that employees were surveyed during a particularly vulnerable period, potentially skewing the results towards identifying urgent needs, especially in mental health and wellbeing improvement measures.

It’s important to note that the employee survey was initially conducted as a pilot study at the Faculty of Human Sciences and may not generalize to the entire university. However, all other survey methods were applied university-wide. Additionally, out of 15 invited participants for the Reflective and Evaluative Instrument (REI), only 4 attended the workshop, with 3 others responding via email. Despite this, valuable insights were gathered from the seven participants.

### Conclusion/Future Directions

Our results confirm that a multidimensional needs assessment is essential for identifying diverse health and wellbeing needs. The comprehensive university employee health needs assessment conducted through the social-ecological framework within the “Healthy University Bern” initiative offers insights into current health challenges and sets a precedent for future health interventions in university settings. Looking ahead practically, the next steps involve developing an action plan, implementing these actions, and evaluating them. From a research perspective, future directions involve conducting regular evaluations of these needs, perhaps every three to 5 years, to track changes and improvements.
