# Supplementary material for: Comprehensive Needs Assessment for Workplace Health Promotion: A Case Study of the University of Bern
Source: Int J Public Health. 2025 Sep 18;70:1608274. doi: 10.3389/ijph.2025.1608274 (PMC12488525; doi:10.3389/ijph.2025.1608274)
Supplement: Supplementary file 1 [file DataSheet1.pdf]

Supplementary Material Tables

Table S1: Analyzed documents of the Policy Analysis (Gesunde UniBE, Switzerland, 2021)

**Main documents**	
**Strategies and mission statement of the university and faculties**	
1.	Strategie 2021 der Universität Bern
2.	Leitbild der Universität Bern
3.	Fakultätsstrategie 2030. Phil. -hum. Fakultät, Universität Bern[1]. (not yet available online)
4.	Medizinische Fakultät der Universität Bern – Strategie 2030[4].
5.	Personalleitbild der Universität Bern.
6.	Leitlinien Kommission für die Gleichstellung für den institutionellen Umgang mit Diversität an der Universität Bern: «Chancengleichheit an der Universität Bern».
7.	Nachhaltigkeitsleitbild der Universität Bern.
8.	Qualitätsstrategie der Universität Bern 2018-2021.
9.	Strategie Unisport der Universität Bern. (not yet available online)
10.	Leitbild Universität Sport.
**Specific documents**	
**Personnel regulations**	
11.	Reglement über die Anstellung an der Universität Bern (Anstellungsreglement) vom 25.01.2019
12.	Reglement über Aufgaben und Anstellung von Postdoktorandinnen und Postdoktoranden vom 16.04.2019
13.	Richtlinien zu Aufgaben und Anstellung von ordentlichen und ausserordentlichen Professorinnen und Professoren an der Universität Bern vom 01.09.2020.
14.	Richtlinien und Aufgaben von Assistenzprofessorinnen und Assistenzprofessoren mit Tenure Track an der Universität Bern vom 01.09.2020.
15.	Richtlinien betreffend Jobsharing für Professuren an der Universität Bern vom 29. Juni 2015.
16.	Reglement über die Jahresabreitszeit vom 01.08.2017.
17.	Informationen über das Arbeitsverhältnis für neue Mitarbeitende, Universität Bern.
18.	Reglement über die Ombudsperson der Universität Bern.
19.	Personal Reglemente Kanton Bern.
20.	Führungswerte Kanton Bern.
**Research and promotion of young scientists Regulations**	
21.	Reglement betreffend Förderungsmittel für die Mobilität von Doktorierenden der Universität Bern (UniBE Doc Mobility») vom 01.12.2020.
22.	Reglement betreffend der Ko-Finanzierung von internationalen Postdoktorierenden mit dem EU Qualitätslabel «Seal of Excellence» («SELF Fund») vom 26.07.2018)
23.	Reglement betreffend der Förderung von Interdisziplinaritäts-Grants an der Universität Bern (UniBE ID Grants») vom 31.07.2015
24.	Reglement der Universitätsleitung betreffend Förderungsmittel für Nachwuchsförderung an der Universität Bern («UniBE Initior Grants») vom 17. 12.2013 mit Änderungen.
25.	Reglement über den Nachwuchsförderungs-Projektpool der Universität Ber vom 25.02.2014.
**Organizational law**	
26.	Geschäftsordnung über die Organisation der Universitätsleitung vom 1. Juni 2018.
27.	Geschäftsordnung der Komission für Nachhaltige Entwicklung der Universität Bern vom 29.05.2012.
28.	Geschäftsordnung der Komission für die Förderung des wissenschaftlichen Nachwuchses der Universität Bern vom 07.06.2018.
29.	Geschäftsordnung der Komission für die Gleichstellung von Frauen und Männern an der Universität Bern vom 15.11.2011.
30.	Geschäftsordnung des Senats an der Universität bern vom 15.04.2014.
31.	Reglement über die Organisation der Philosphisch-humanwissenschaftlichen Fakultät der Universität Bern (Fakultätsreglement, FaR Phil.- hum.) vom 22.09.2014.
**Equal opportunities and equality**	
32.	Charta «Familie in der Hochschule «Vereinbarkeit (2016). Charta: Familie in der Hochschule. Universität Bern.
33.	Reglement betreffend Förderungsmittel für einen 120%-Care-Grant für Postdoktorandinnen und Postdoktoranden an der Universität Bern vom 01.04.2020.
34.	Leitlinien zur Ausrichtung von Beiträgen aus dem Entlastungspool für Professorinnen und Professoren mit Betreuungspflichten und Jobsharing-Tandems vom 04.12.2018.
**Sustainability**	
35.	Nachhaltige Entwicklung an der Universität Bern Bericht zum Nachhaltigkeitsmonitoring 2015.
**Gastronomy**	
36.	Gastronomie der Universität Bern

[1] The other departments do not have a faculty strategy and may design one in the future.

Table S2: Selected variables of the 2nd part of the employee survey : Health determinant-environment (Gesunde UniBE, Switzerland, 2021)

			Total sample			Descriptive Demography		
**Area**		**Variable**	**N**	**M**	**SD**	**Gender difference**	**Employment relationship**	**Differences in function (7 groups of people)**
Workload	84	Time Pressure<br>(scale 1-5)<br>1= very rarely/never<br>5= very often/permanently	182	3.16	0.96			* Auxiliary Assistants < all other groups of people<br>*Administrative and Technical Staff < Professors, Lecturers, Postdocs. Ph.D. Students
	84	Qualitative Overload<br>(scale 1-5)<br>1= very rarely/never<br>5= very often/permanently	182	2.32	0.89		* Temporary > Permanent	* Ph.D. Students > Professors, Lecturers, Research Assistants, Administrative and Technical Stuff, Auxiliary Assistants<br>*Postdocs > Administrative and Technical Stuff, Auxiliary Assistants
		Performance Constraints<br>(scale 1-5)<br>1= does not apply at all<br>2= strongly agree	181	4.25	0.80			
Social Work Conditions	84	Social Stressors Supervisors<br>(Scale 1-5)<br>1= does not apply<br>5= is completely true	163	1.35	0.63			
	84	Social stressors Work colleagues<br>(scale 1-5)<br>1= does not apply<br>5= completely true	181	1.30	0.54			
Job Resources	84	Scope of action<br>(scale 1-5)<br>1= very little<br>5= very much	182	4.18	0.61			* Auxiliary Assistants, Ph.D. Students < Lecturers, Postdocs<br>*Postdocs > Research Assistants, Administrative and Technical Stuff

Work and Management Culture	84	Supportive Supervisor Behaviour (scale 1-5) 1= does not apply 5= completely true	162	3.9	0.87			*Auxiliary Assistants > Lecturers, Research Assistants, Ph.D. Students *Lecturers < Administrative and Technical Stuff
		Feedback/Collaboration (scale 1-5) 1= Does not apply at all 5= strongly agree	156	3.86	1.01			
		Psychological Safety (scale 1-5) 1= not at all true 5= fully applies	180	4.00	0.64			
Organizational Work Conditions	84	Job Insecurity (scale 1-5) 1= do not agree at all 5= completely agree	176	2.22	1.11		* Temporary > Permanent	*Professors < Postdocs, Research Assistants, Ph.D. Students, Auxiliary Assistants, Administrative and Technical Stuff *Lecturers < Postdocs, Research Assistants, Ph.D. Students, Auxiliary Assistants *Postdocs < Administrative and Technical Stuff
		Development Opportunities and Career Prospects (scale 1-5) 1= does not apply at all 5= very true	181	3.71	0.86			
		Traceability of the Temporary Employment (scale 1-5) 1= does not apply at all 5= very true	117	2.91	1.36			* Postdocs > Ph.D. Students, Auxiliary Assistants *Administrative and Technical Staff < Ph.D. Students, Auxiliary Assistants *Research Assistants < Auxiliary Assistants
Work-Life Interference		Reconciliation of Work and Family/Private Life (scale 1-5) 1= does not apply 5= completely true	180	3.91	0.73			

*Notes. N = sample size. M = means. SD = standard deviation. * = Significant post hoc levels for multiple comparisons p<0.05.*

Legend:

Results worse than in comparison samples/guidelines (84).	Results same as in comparison samples/guidelines (84).	Results better than in comparison samples/guidelines(84).

Table S3: Selected variables of the 3rd part of the employee survey: Health determinant-person (Gesunde UniBE, Switzerland, 2021)

			Total sample				Differentiation according to 7 groups of people		
**Area**		**Variable**	**N**	**%**	**M**	**SD**	**Gender difference**	**Employment relationship**	**Differences in function**
Personal Resources	85	Self-efficacy (scale 1-5) 1= does not apply 5= completely true	181		4.13	0.51			
		Self-regulation (scale 1-4) 1= not true 4= true exactly	181		3.13	0.55			
Health Behaviour	86	Fruit and Vegetable Consumption (Serving of fruits and vegetables per day)[2]	180		3.71	1.61	f > m Trend towards significance (p=0.05)		
	87	Strenuous and moderate Activity per Week (MVPA) [min/week].	181		274.10	223.40			
		Walking [min/week]	178		183.50	185.50			
	85	Sitting Behaviour (hours/day)	167		8.10	2.70			* Postdocs > Lecturers, Auxiliary Assistants, Administrative Technical Stuff *Ph.D. Students > Lecturers
		< than 1 time per Month Excessive Alcohol Consumption[3]	4	2.2					
	88	Occasional Tobacco Use	13	7.1					

[2] One serving is about a handful or 120 g.

[3] One standard alcoholic drink is equivalent to about 3dl of beer (5% by volume), 1dl of wine or sparkling wine (12.5% by volume), 2cl of hard liquor (55% by volume) or 4cl of liqueur (30% by volume). Excessive alcohol consumption equals women < 6 standard alcoholic beverages, men < 8 standard alcoholic beverages.

88	Regular Tobacco Use	9	4.9

*Notes. N = sample size. M = means. SD = standard deviation. * = Significant post hoc levels for multiple comparisons p<0.05.*

Legend:

Results worse than in comparison samples/guidelines (85).	Results same as in comparison samples/guidelines (85).	Results better than in comparison samples/guidelines (85, 86, 88).

Table S1: Selected variables of the 4th part of the employee survey: Health indicators (Gesunde UniBE, Switzerland, 2021)

			**Total sample**			**Differentiation according to 7 groups of people**		
**Area**		**Variable**	**N**	**M**	**SD**	**Gender difference**	**Employment relationship**	**Differences in function**
Physical Health	89	Overweight (25 < BMI)	33					
Mental Health	90	Work-Related emotional Exhaustion<br>(scale 1-4)<br>1=completely inapplicable<br>4= completely true	181	2.23	0.51			*Professors < Postdocs, Ph.D. Students, Research Assistants<br>*Auxiliary assistants < Ph.D. Students, Research Assistants
	91	Perceived Stress Symptoms<br>(Sum score of 4 items 0-16)<br>0= never<br>4= very often	180	5.67	2.83		* Temporary > Permanent	*Professors < Ph.D. Students, Auxiliary Assistants<br>Lecturers < Auxiliary Assistants
Wellbeing		WHO-Five: Well-Being Index<br>(Sum score of 5 items 1-30)<br>(scale 1-6)<br>1= at no time<br>6= all the time	180	14.26	4.77		* Temporary > Permanent	

*Notes. N = sample size. M = means. SD = standard deviation. * = Significant post hoc levels for multiple comparisons p<0.05.*

Legend:

Results worse than in comparison samples/guidelines (90).	Results same as in comparison samples/guidelines (91).	Results better than in comparison samples/guidelines (89).

Table S2: Selected variables of the 5th part of the employee survey: Organizational indicators (Gesunde UniBE, Switzerland, 2021)

			Total sample				Differentiation according to 7 groups of people		
**Area**		**Variable**	**N**	**%**	**M**	**SD**	**Gender difference**	**Employment relationship**	**Differences in function**
Attitudes Toward Work and Work Engagement	84	General Job Satisfaction (scale 1-7) 1= exceptionally dissatisfied 7= exceptionally satisfied	181	100	5.08	1.31			
	84	Work Engagement (scale 1-7) 1= never 7= always/every day	179	100	3.77	1.11			*Professors, Postdocs > Research Assistants, Administrative and Technical Stuff, Auxiliary Assistants *Lecturers > Auxiliary Assistants
Performance	92	Presentism (in % of working time)	180	12.3	12.25	0.17			
	92	Absentism (in % of working time)	180	2.4	2.37	0.15			

*Notes. N = sample size. M = means. SD = standard deviation. * = Significant post hoc levels for multiple comparisons p<0.05.*

Legend:

Results worse than in comparison samples/guidelines (84, 92).	Results same as in comparison samples/guidelines.	Results better than in comparison samples/guidelines (92).

Table S6: Statistical findings on variables of the Employee Survey (All variables except for «Number of Sedentary Hours per Day» are normally distributed. In all analyses of variance, the tests of homogenity are non-significant (p < .05). The significance level was set at .05.) (Gesunde UniBE, Switzerland, 2021)

Variance analysis – Gender

	Descriptive statistics						ANOVA		Effect size[ab]
		N	M	SD		df	F	Sig.	η²
Scale Qualitative Overload	Male	71	2.20	.83	Between Groups	1	1.68	.20	.01
	Female	102	2.37	.91	Within Groups	171			
	Total	173	2.30	.88	Total	172			
Scale of Closeness	Male	71	4.22	.58	Between Groups	1	.43	.51	.00
	Female	102	4.16	.61	Within Groups	171			
	Total	173	4.19	.60	Total	172			
Scale Workplace Insecurity	Male	67	2.25	1.19	Between Groups	1	.24	.63	.00
	Female	100	2.17	1.02	Within Groups	165			
	Total	167	2.20	1.09	Total	166			
Traceability of the Temporary Employment	Male	38	3.74	1.31	Between Groups	1	.79	.38	.01
	Female	73	3.96	1.22	Within Groups	109			
	Total	111	3.88	1.25	Total	110			
Consumed Number of Portions of Fruits and Vegetables per Day	Male	71	3.39	1.58	Between Groups	1	4.06	.05	.02
	Female	101	3.90	1.66	Within Groups	170			
	Total	172	3.69	1.64	Total	171			
Scale of Work Engagement	Male	71	3.85	1.13	Between Groups	1	.68	.41	.00
	Female	101	3.71	1.12	Within Groups	170			
	Total	172	3.77	1.12	Total	171			
Scale of Work-Related Emotional Exhaustion	Male	71	2.15	.52	Between Groups	1	2.31	.13	.01
	Female	101	2.27	.51	Within Groups	170			
	Total	172	2.22	.51	Total	171			

Scale of Perceived Stress Symptoms	Male	71	1.28	.67	Between Groups	1	3.33	.07	.02
	Female	101	1.48	.73	Within Groups	170			
	Total	172	1.40	.71	Total	171			
WHO-Five Well-Being Index	Male	70	14.80	4.84	Between Groups	1	1.09	.30	.01
	Female	102	14.03	4.70	Within Groups	170			
	Total	172	14.34	4.76	Total	171			

a. Eta-squared is estimated based on the model with fixed effects.

b. Negative, yet less biased estimates are retained, not rounded up to zero.

Mann-Whitney- U-Test - Gender

	Descriptive statistics			Ranks				Test statistics			Effect size
	N	M	SD	Gender	N	Mean Rank	Sum of Ranks	U	Z	Asymp. Sig. (2-sided)	r
Scale of Time Pressure	173	3.15	.97	Male	71	89.23	6335.00	3463.00	-.49	.62	.04
				Female	102	85.45	8716.00				
				Total	173						
Number of Sedentary Hours per Day	171	8.15	3.42	Male	70	84.96	5947.00	3462.00	-.23	.82	.02
				Female	101	86.72	8759.00				
				Total	171						
Scale of Supportive Supervisory Behavior	153	3.91	.85	Male	55	80.19	4410.50	2519.50	-.67	.50	.06
				Female	98	75.21	7370.50				
				Total	153						

Variance analysis - Employment Relationship

Descriptive statistics					ANOVA				Effect size[ab]
		N	M	SD		df	F	Sig.	η²
Scale Qualitative Overload	Temporary	117	2.45	.88	Between Groups	1	7.58	.01	.04
	Permanent	65	2.08	.88	Within Groups	180			
	Total	182	2.32	.89	Total	181			
Consumed Number of Portions of Fruits and Vegetables per Day	Temporary	116	3.83	1.73	Between Groups	1	1.80	.18	.01
	Permanent	64	3.49	1.35	Within Groups	178			
	Total	180	3.71	1.61	Total	179			
Scale of Work Engagement	Temporary	115	3.65	1.05	Between Groups	1	3.64	.06	.02
	Permanent	64	3.98	1.18	Within Groups	177			
	Total	179	3.77	1.11	Total	178			
WHO-Five Well-Being Index	Temporary	116	13.54	4.68	Between Groups	1	7.54	.01	.04
	Permanent	64	15.55	4.71	Within Groups	178			
	Total	180	14.26	4.77	Total	179			
Scale of Time Pressure	Temporary	117	3.12	1.01	Between Groups	1	.51	.48	.00
	Permanent	65	3.22	.86	Within Groups	180			
	Total	182	3.16	.96	Total	181			
Scale of Supportive Supervisory Behavior	Temporary	113	3.95	.84	Between Groups	1	1.35	.25	.01
	Permanent	49	3.78	.94	Within Groups	160			
	Total	162	3.90	.87	Total	161			

a. Eta-squared is estimated based on the model with fixed effects.

b. Negative, yet less biased estimates are retained, not rounded up to zero.

Mann-Whitney U-Test - Employment Relationship

	Descriptive Statistics			Ranks				Test statistics			Effect size
	N	M	SD	Employment relationship	N	Mean Rank	Sum of Ranks	U	Z	Asymp. Sig. (2-sided)	r
Scale of Closeness	182	4.18	.61	Temporary	117	88.10	10307.50	3404.50	-1.18	.24	.09
				Permanent	65	97.62	6345.50				
				Total	182						
Scale Workplace Insecurity	176	2.22	1.11	Temporary	114	103.89	11843.00	1780.00	-5.48	<.001	.41
				Permanent	62	60.21	3733.00				
				Total	176						
Number of Sedentary Hours per Day	180	8.20	3.39	Temporary	115	94.90	10913.00	3232.00	-1.52	.13	.11
				Permanent	65	82.72	5377.00				
				Total	180						
Scale of Perceived Stress Symptoms	181	1.42	.71	Temporary	116	101.19	11738.00	2588.00	-3.52	<.001	.26
				Permanent	65	72.82	4733.00				
				Total	181						
Scale of Work-Related Emotional Exhaustion	181	2.23	.51	Temporary	116	96.45	11188.00	3138	-1.87	.06	.14
				Permanent	65	81.28	5283.00				
				Total	181						

Variance analysis - Function

		Descriptive statistics				ANOVA			Effect size[ab]
		N	M	SD		df	F	Sig.	η²
Scale Qualitative Overload	Professor	15	2.24	.87	Between Groups	6	4.30	<.001	.14
	Lecturer	16	2.29	.76	Within Groups	162			
	Postdoctoral Researcher	16	2.60	.98	Total	168			
	Ph.D. Student	38	2.81	.86					
	Research Assistant	26	2.23	.86					
	Administrative and Technical Stuff	24	1.92	.78					
	Auxiliary Assistant	34	2.01	.75					
	Total	169	2.31	.88					
Traceability of the Temporary Employment	Postdoctoral Researcher	15	3.13	1.30	Between Groups	4	4.28	<.001	.14
	Ph.D. Student	37	4.03	1.19	Within Groups	107			
	Research Assistant	23	3.61	1.34	Total	111			
	Administrative and Technical Stuff	3	2.33	2.31					
	Auxiliary Assistant	34	4.32	.94					
	Total	112	3.87	1.27					
Consumed Number of Portions of Fruits and Vegetables per Day	Professor	15	3.80	1.90	Between Groups	6	.60	.73	.02
	Lecturer	16	3.13	.81	Within Groups	161			
	Postdoctoral Researcher	16	3.59	1.93	Total	167			
	Ph.D. Student	38	3.87	1.81					
	Research Assistant	25	3.88	1.48					
	Administrative and Technical Stuff	24	3.52	1.39					
	Auxiliary Assistant	34	3.93	1.79					
	Total	168	3.73	1.64					

Scale of Work Engagement	Professor	15	4.33	1.12	Between Groups	6	2.90	.01	.10
	Lecturer	16	4.08	1.09	Within Groups	161			
	Postdoctoral Researcher	16	4.31	.82	Total	167			
	Ph.D. Student	37	3.70	1.02					
	Research Assistant	26	3.62	.99					
	Administrative and Technical Stuff	24	3.47	1.43					
	Auxiliary Assistant	34	3.33	.99					
	Total	168	3.73	1.11					
Scale of Work-Related Emotional Exhaustion	Professor	15	1.97	.437	Between Groups	6	2.26	.04	.08
	Lecturer	16	2.22	.420	Within Groups	161			
	Postdoctoral Researcher	16	2.36	.581	Total	167			
	Ph.D. Student	38	2.37	.595					
	Research Assistant	26	2.36	.554					
	Administrative and Technical Stuff	24	2.14	.463					
	Auxiliary Assistant	33	2.07	.435					
	Total	168	2.22	.523					
Scale of Perceived Stress Symptoms	Professor	15	1.00	.42	Between Groups	6	2.61	.02	.09
	Lecturer	16	1.17	.49	Within Groups	161			
	Postdoctoral Researcher	16	1.41	.66	Total	167			
	Ph.D. Student	38	1.61	.81					
	Research Assistant	26	1.38	.67					
	Administrative and Technical Stuff	24	1.31	.59					
	Auxiliary Assistant	33	1.69	.83					
	Total	168	1.43	.72					

a. Eta-squared is estimated based on the model with fixed effects.

b. Negative, yet less biased estimates are retained, not rounded up to zero.

Multiple Comparisons – Function

LSD

Dependent Variable	(I) FUNCTION	(J) FUNCTION	Mean difference (I-J)	SE	Sig.	95% Confidence Interval	
						Lover Bound	Upper Bound
Scale Qualitative Overload	Professor	Lecturer	-.05	.30	.87	-.64	.54
		Postdoctoral Researcher	-.36	.30	.23	-.95	.23
		Ph.D. Student	-.56*	.25	.03	-1.06	-.06
		Research Assistant	.01	.27	.96	-.52	.55
		Administrative and Technical Stuff	.33	.27	.23	-.21	.87
		Auxiliary Assistant	.23	.26	.36	-.27	.74
	Lecturer	Professor	.05	.30	.87	-.54	.64
		Postdoctoral Researcher	-.31	.29	.29	-.89	.27
		Ph.D. Student	-.52*	.25	.04	-1.00	-.03
		Research Assistant	.06	.26	.82	-.46	.58
		Administrative and Technical Stuff	.38	.27	.16	-.15	.90
		Auxiliary Assistant	.28	.25	.26	-.22	.78
	Postdoctoral Researcher	Professor	.36	.30	.23	-.23	.95
		Lecturer	.31	.29	.29	-.27	.89
		Ph.D. Student	-.20	.25	.41	-.69	.29
		Research Assistant	.37	.26	.16	-.15	.89
		Administrative and Technical Stuff	.69*	.27	.01	.16	1.22
		Auxiliary Assistant	.59*	.25	.02	.10	1.09
	Ph.D. Student	Professor	.56*	.25	.03	.06	1.06
		Lecturer	.52*	.25	.04	.03	1.00

		Postdoctoral Researcher	.20	.25	.41	-.29	.69
		Research Assistant	.58*	.21	.01	.16	.99
		Administrative and Technical Stuff	.89*	.22	<.001	.46	1.32
		Auxiliary Assistant	.80*	.20	<.001	.41	1.18
	Research Assistant	Professor	-.01	.27	.96	-.55	.52
		Lecturer	-.06	.26	.82	-.58	.46
		Postdoctoral Researcher	-.37	.26	.16	-.89	.15
		Ph.D. Student	-.58*	.21	.01	-.99	-.16
		Administrative and Technical Stuff	.31	.24	.18	-.15	.78
		Auxiliary Assistant	.22	.22	.31	-.21	.65
	Administrative and Technical Stuff	Professor	-.33	.27	.23	-.87	.21
		Lecturer	-.37	.27	.16	-.90	.15
		Postdoctoral Researcher	-.69*	.27	.01	-1.22	-.16
		Ph.D. Student	-.89*	.22	<.001	-1.32	-.46
		Research Assistant	-.31	.24	.18	-.78	.15
		Auxiliary Assistant	-.09	.22	.67	-.53	.34
	Auxiliary Assistant	Professor	-.23	.26	.36	-.74	.27
		Lecturer	-.28	.25	.26	-.78	.22
		Postdoctoral Researcher	-.59*	.25	.02	-1.09	-.10
		Ph.D. Student	-.80*	.20	<.001	-1.18	-.41
		Research Assistant	-.22	.22	.31	-.65	.21
		Administrative and Technical Stuff	.09	.22	.67	-.34	.53
Consumed Number of Portions of Fruits and Vegetables per Day	Professor	Lecturer	.67	.59	.26	-.50	1.85
		Postdoctoral Researcher	.21	.59	.73	-.97	1.38
		Ph.D. Student	-.07	.50	.89	-1.06	.93
		Research Assistant	-.08	.54	.88	-1.15	.99

	Administrative and Technical Stuff	.28	.54	.61	-.80	1.35
	Auxiliary Assistant	-.13	.51	.81	-1.14	.89
Lecturer	Professor	-.67	.59	.26	-1.85	.50
	Postdoctoral Researcher	-.47	.58	.42	-1.62	.69
	Ph.D. Student	-.74	.49	.13	-1.72	.23
	Research Assistant	-.75	.53	.16	-1.80	.29
	Administrative and Technical Stuff	-.40	.53	.46	-1.45	.66
	Auxiliary Assistant	-.80	.50	.11	-1.79	.19
Postdoctoral Researcher	Professor	-.21	.59	.73	-1.38	.97
	Lecturer	.47	.58	.42	-.69	1.62
	Ph.D. Student	-.27	.49	.58	-1.25	.70
	Research Assistant	-.29	.53	.59	-1.33	.76
	Administrative and Technical Stuff	.07	.53	.89	-.98	1.13
	Auxiliary Assistant	-.33	.50	.51	-1.32	.66
Ph.D. Student	Professor	.07	.50	.89	-.93	1.06
	Lecturer	.74	.49	.13	-.23	1.72
	Postdoctoral Researcher	.27	.49	.58	-.70	1.25
	Research Assistant	-.01	.43	.98	-.85	.83
	Administrative and Technical Stuff	.35	.43	.42	-.50	1.20
	Auxiliary Assistant	-.06	.39	.88	-.83	.71
Research Assistant	Professor	.08	.54	.88	-.99	1.15
	Lecturer	.75	.53	.16	-.29	1.80
	Postdoctoral Researcher	.29	.53	.59	-.76	1.33
	Ph.D. Student	.01	.43	.98	-.83	.85
	Administrative and Technical Stuff	.36	.47	.45	-.57	1.29
	Auxiliary Assistant	-.05	.44	.92	-.91	.81

	Administrative and Technical Stuff	Professor	-.28	.54	.61	-1.35	.80
		Lecturer	.40	.53	.46	-.66	1.45
		Postdoctoral Researcher	-.07	.53	.89	-1.13	.98
		Ph.D. Student	-.35	.43	.42	-1.20	.50
		Research Assistant	-.36	.47	.45	-1.29	.57
		Auxiliary Assistant	-.41	.44	.36	-1.28	.46
	Auxiliary Assistant	Professor	.13	.51	.81	-.89	1.14
		Lecturer	.80	.50	.11	-.19	1.79
		Postdoctoral Researcher	.33	.50	.51	-.66	1.32
		Ph.D. Student	.06	.39	.88	-.71	.83
		Research Assistant	.05	.44	.92	-.81	.91
		Administrative and Technical Stuff	.41	.44	.36	-.46	1.28
Scale of Work Engagement	Professor	Lecturer	.25	.39	.52	-.51	1.01
		Postdoctoral Researcher	.02	.39	.96	-.74	.79
		Ph.D. Student	.63	.33	.06	-.02	1.28
		Research Assistant	.72*	.35	.04	.03	1.41
		Administrative and Technical Stuff	.86*	.35	.02	.16	1.56
		Auxiliary Assistant	1.00*	.33	<.001	.34	1.66
	Lecturer	Professor	-.25	.39	.52	-1.01	.51
		Postdoctoral Researcher	-.23	.38	.55	-.98	.52
		Ph.D. Student	.38	.32	.24	-.26	1.02
		Research Assistant	.47	.34	.17	-.21	1.14
		Administrative and Technical Stuff	.61	.35	.08	-.08	1.30
		Auxiliary Assistant	.75*	.33	.02	.10	1.40
	Postdoctoral Researcher	Professor	-.02	.39	.96	-.79	.74
		Lecturer	.23	.38	.55	-.52	.98

	Ph.D. Student	.61	.32	.06	-.03	1.25
	Research Assistant	.70*	.34	.04	.02	1.37
	Administrative and Technical Stuff	.84*	.35	.02	.15	1.53
	Auxiliary Assistant	.98*	.33	<.001	.33	1.62
Ph.D. Student	Professor	-.63	.33	.06	-1.28	.02
	Lecturer	-.38	.32	.24	-1.02	.26
	Postdoctoral Researcher	-.61	.32	.06	-1.25	.03
	Research Assistant	.09	.28	.75	-.46	.63
	Administrative and Technical Stuff	.23	.28	.42	-.33	.79
	Auxiliary Assistant	.37	.26	.15	-.14	.87
Research Assistant	Professor	-.72*	.35	.04	-1.41	-.03
	Lecturer	-.47	.34	.17	-1.14	.21
	Postdoctoral Researcher	-.70*	.34	.04	-1.37	-.02
	Ph.D. Student	-.09	.28	.75	-.63	.46
	Administrative and Technical Stuff	.14	.31	.64	-.46	.75
	Auxiliary Assistant	.28	.28	.32	-.27	.84
Administrative and Technical Stuff	Professor	-.86*	.35	.02	-1.56	-.16
	Lecturer	-.61	.35	.08	-1.30	.08
	Postdoctoral Researcher	-.84*	.35	.02	-1.53	-.15
	Ph.D. Student	-.23	.28	.42	-.79	.33
	Research Assistant	-.14	.31	.64	-.75	.46
	Auxiliary Assistant	.14	.29	.63	-.43	.71
Auxiliary Assistant	Professor	-1.00*	.33	<.001	-1.66	-.34
	Lecturer	-.75*	.33	.02	-1.40	-.10
	Postdoctoral Researcher	-.98*	.33	<.001	-1.62	-.33
	Ph.D. Student	-.37	.26	.15	-.87	.14

		Research Assistant	-.28	.28	.32	-.84	.27
		Administrative and Technical Stuff	-.14	.29	.63	-.71	.43
Scale of Work-Related Emotional Exhaustion	Professor	Lecturer	-.25	.18	.17	-.62	.11
		Postdoctoral Researcher	-.39*	.18	.03	-.75	-.03
		Ph.D. Student	-.40*	.16	.01	-.71	-.10
		Research Assistant	-.40*	.17	.02	-.72	-.07
		Administrative and Technical Stuff	-.17	.17	.31	-.51	.16
		Auxiliary Assistant	-.10	.16	.52	-.42	.21
	Lecturer	Professor	.25	.18	.17	-.11	.62
		Postdoctoral Researcher	-.14	.18	.45	-.50	.22
		Ph.D. Student	-.15	.15	.33	-.45	.15
		Research Assistant	-.14	.16	.38	-.46	.18
		Administrative and Technical Stuff	.08	.17	.63	-.25	.41
		Auxiliary Assistant	.15	.16	.34	-.16	.46
	Postdoctoral Researcher	Professor	.39*	.18	.03	.03	.75
		Lecturer	.14	.18	.45	-.22	.50
		Ph.D. Student	-.01	.15	.94	-.31	.29
		Research Assistant	.00	.16	.98	-.32	.32
		Administrative and Technical Stuff	.22	.17	.19	-.11	.54
		Auxiliary Assistant	.29	.16	.07	-.02	.60
	Ph.D. Student	Professor	.40*	.16	.01	.10	.71
		Lecturer	.15	.15	.33	-.15	.45
		Postdoctoral Researcher	.01	.15	.94	-.29	.31
		Research Assistant	.01	.13	.95	-.25	.27
		Administrative and Technical Stuff	.23	.13	.09	-.03	.49
		Auxiliary Assistant	.30*	.12	.01	.06	.54

	Research Assistant	Professor	.40*	.17	.02	.07	.72
		Lecturer	.14	.16	.38	-.18	.46
		Postdoctoral Researcher	.00	.16	.98	-.32	.32
		Ph.D. Student	-.01	.13	.95	-.27	.25
		Administrative and Technical Stuff	.22	.14	.13	-.06	.51
		Auxiliary Assistant	.29*	.13	.03	.03	.56
	Administrative and Technical Stuff	Professor	.17	.17	.31	-.16	.51
		Lecturer	-.08	.17	.63	-.41	.25
		Postdoctoral Researcher	-.22	.17	.19	-.54	.11
		Ph.D. Student	-.23	.13	.09	-.49	.03
		Research Assistant	-.22	.14	.13	-.51	.06
		Auxiliary Assistant	.07	.14	.61	-.20	.34
	Auxiliary Assistant	Professor	.10	.16	.52	-.21	.42
		Lecturer	-.15	.16	.34	-.46	.16
		Postdoctoral Researcher	-.29	.16	.07	-.60	.02
		Ph.D. Student	-.30*	.12	.01	-.54	-.06
		Research Assistant	-.29*	.13	.03	-.56	-.03
		Administrative and Technical Stuff	-.07	.14	.61	-.34	.20
Scale of Perceived Stress Symptoms	Professor	Lecturer	-.17	.25	.49	-.67	.32
		Postdoctoral Researcher	-.41	.25	.11	-.90	.09
		Ph.D. Student	-.61*	.21	<.001	-1.02	-.19
		Research Assistant	-.38	.23	.09	-.83	.06
		Administrative and Technical Stuff	-.31	.23	.18	-.77	.14
		Auxiliary Assistant	-.69*	.22	<.001	-1.12	-.26
	Lecturer	Professor	.17	.25	.49	-.32	.67
		Postdoctoral Researcher	-.23	.25	.34	-.72	.25

	Ph.D. Student	-.43*	.21	.04	-.84	-.02
	Research Assistant	-.21	.22	.34	-.65	.22
	Administrative and Technical Stuff	-.14	.22	.53	-.58	.30
	Auxiliary Assistant	-.52*	.21	.02	-.94	-.10
Postdoctoral Researcher	Professor	.41	.25	.11	-.09	.90
	Lecturer	.23	.25	.34	-.25	.72
	Ph.D. Student	-.20	.21	.34	-.61	.21
	Research Assistant	.02	.22	.92	-.42	.46
	Administrative and Technical Stuff	.09	.22	.68	-.35	.54
	Auxiliary Assistant	-.28	.21	.18	-.70	.14
Ph.D. Student	Professor	.61*	.21	<.001	.19	1.02
	Lecturer	.43*	.21	.04	.02	.84
	Postdoctoral Researcher	.20	.21	.34	-.21	.61
	Research Assistant	.22	.18	.22	-.13	.57
	Administrative and Technical Stuff	.29	.18	.11	-.07	.65
	Auxiliary Assistant	-.08	.17	.61	-.41	.24
Research Assistant	Professor	.38	.23	.09	-.06	.83
	Lecturer	.21	.22	.34	-.22	.65
	Postdoctoral Researcher	-.02	.22	.92	-.46	.42
	Ph.D. Student	-.22	.18	.22	-.57	.13
	Administrative and Technical Stuff	.07	.20	.72	-.32	.46
	Auxiliary Assistant	-.30	.18	.10	-.67	.06
Administrative and Technical Stuff	Professor	.31	.23	.18	-.14	.77
	Lecturer	.14	.22	.53	-.30	.58
	Postdoctoral Researcher	-.09	.22	.68	-.54	.35
	Ph.D. Student	-.29	.18	.11	-.65	.07

	Research Assistant	-.07	.20	.72	-.46	.32
	Auxiliary Assistant	-.38*	.19	.05	-.75	-.01
Auxiliary Assistant	Professor	.69*	.22	<.001	.26	1.12
	Lecturer	.52*	.21	.02	.10	.94
	Postdoctoral Researcher	.28	.21	.18	-.14	.70
	Ph.D. Student	.08	.17	.61	-.24	.41
	Research Assistant	.30	.18	.10	-.06	.67
	Administrative and Technical Stuff	.38*	.19	.05	.01	.75

*. The mean difference is significant at the 0.05 level.

Kruskal-Wallis-Test - Function

	Descriptive statistics			Ranks			Test statistics			Effect size
	N	M	SD	Function	N	Mean Rank	Z[a]	df	Asymp. Sig.[b]	ε²
Scale of Closeness	182	4.18	.61	Professor	15	99.90	20.54	6	<.001	.09
				Lecturer	16	104.78				
				Postdoctoral Researcher	16	123.38				
				Ph.D. Student	38	75.09				
				Research Assistant	26	83.42				
				Administrative and Technical Stuff	24	79.81				
				Auxiliary Assistant	34	67				
				Total	169					
Scale of Supportive Supervisory Behavior	162	3.90	.87	Professor	Nc.	Nc.	14.36	6	.03	.06
				Lecturer	15	49.60				
				Postdoctoral Researcher	16	78.34				
				Ph.D. Student	36	70.18				
				Research Assistant	26	68.02				
				Administrative and Technical Stuff	23	82.17				
				Auxiliary Assistant	34	95.21				
				Total	151					
Scale Workplace Insecurity	176	2.22	1.11	Professor	14	41.29	29.03	6	<.001	.15
				Lecturer	15	48.03				
				Postdoctoral Researcher	16	110.16				
				Ph.D. Student	35	86.33				
				Research Assistant	26	100.73				
				Administrative and Technical Stuff	24	77.69				
				Auxiliary Assistant	33	84.85				

				Total	163					
Number of Sedentary Hours per Day	180	8.20	3.39	Professor	15	81.30	13.42	6	.04	.05
				Lecturer	16	60.97				
				Postdoctoral Researcher	16	109.97				
				Ph.D. Student	37	98.45				
				Research Assistant	26	77.40				
				Administrative and Technical Stuff	24	74.71				
				Auxiliary Assistant	33	79.56				
				Total	167					
Scale of Perceived Stress Symptoms	181	1.42	.71	Professor	15	53.43	13.42	6	.04	.05
				Lecturer	16	68.44				
				Postdoctoral Researcher	16	86.56				
				Ph.D. Student	38	94.49				
				Research Assistant	26	82.75				
				Administrative and Technical Stuff	24	78.25				
				Auxiliary Assistant	33	99.83				
				Total	168					
WHO-Five Well-Being Index	180	14.26	4.77	Professor	15	108.93	11.49	6	.07	.03
				Lecturer	16	102.53				
				Postdoctoral Researcher	16	77.13				
				Ph.D. Student	38	77.62				
				Research Assistant	26	93.35				
				Administrative and Technical Stuff	24	88.83				
				Auxiliary Assistant	34	69.06				
				Total	169					

a. The test statistic is adjusted for ties.

b. The significance level is .05

Pairwise Comparisons of Function – Scale of Closeness

Sample 1-Sample 2	Test statistic	SE	Standard test statistic	Sig.	Ad. sig.[a]
Auxiliary Assistant-Ph.D. Student	8.09	11.49	.70	.48	1.00
Auxiliary Assistant-Administrative and Technical Stuff	12.81	12.97	.99	.32	1.00
Auxiliary Assistant-Research Assistant	16.42	12.68	1.30	.20	1.00
Auxiliary Assistant-Professor	32.90	15.08	2.18	.03	.61
Auxiliary Assistant-Lecturer	37.78	14.75	2.56	.01	.22
Auxiliary Assistant-Postdoctoral Researcher	56.38	14.75	3.82	<.001	.00
Ph.D. Student-Administrative and Technical Stuff	-4.72	12.69	-.37	.71	1.00
Ph.D. Student-Research Assistant	-8.33	12.38	-.67	.50	1.00
Ph.D. Student-Professor	24.81	14.84	1.67	.09	1.00
Ph.D. Student-Lecturer	29.69	14.50	2.05	.04	.85
Ph.D. Student-Postdoctoral Researcher	48.28	14.50	3.33	<.001	.02
Administrative and Technical Stuff-Research Assistant	3.61	13.77	.26	.79	1.00
Administrative and Technical Stuff-Professor	20.09	16.01	1.25	.21	1.00
Administrative and Technical Stuff-Lecturer	24.97	15.70	1.59	.11	1.00
Administrative and Technical Stuff-Postdoctoral Researcher	43.56	15.70	2.77	.01	.12

Research Assistant-Professor	16.48	15.78	1.04	.30	1.00
Research Assistant-Lecturer	21.36	15.46	1.38	.17	1.00
Research Assistant-Postdoctoral Researcher	39.95	15.46	2.58	.01	.20
Professor-Lecturer	-4.88	17.49	-.28	.78	1.00
Professor-Postdoctoral Researcher	-23.47	17.49	-1.34	.18	1.00
Lecturer-Postdoctoral Researcher	-18.59	17.20	-1.08	.28	1.00

Each row tests the null hypothesis that the distributions in sample 1 and sample 2 are equal. Asymptotic significances (2-sided tests) are displayed. The significance level is .05.

a. Significance values are adjusted by the Bonferroni correction for multiple tests.

Pairwise Comparisons of Function – Scale of Supportive Supervisory Behavior

Sample 1-Sample 2	Test statistic	SE	Standard test statistic	Sig.	Ad. sig.[a]
Lecturer-Professor	6.90	44.96	.15	.88	1.00
Lecturer-Research Assistant	-18.42	14.11	-1.31	.19	1.00
Lecturer-Ph.D. Student	-20.58	13.38	-1.54	.12	1.00
Lecturer-Postdoctoral Researcher	-28.74	15.64	-1.84	.07	1.00
Lecturer-Administrative and Technical Stuff	-32.57	14.45	-2.25	.02	.51
Lecturer- Auxiliary Assistant	-45.61	13.49	-3.38	<.001	.02
Professor-Research Assistant	-11.52	44.36	-.26	.80	1.00
Professor-Ph.D. Student	-13.68	44.13	-.31	.76	1.00
Professor-Postdoctoral Researcher	-21.84	44.87	-.49	.63	1.00
Professor-Administrative and Technical Stuff	-25.67	44.47	-.58	.56	1.00
Professor- Auxiliary Assistant	-38.71	44.17	-.88	.38	1.00
Research Assistant-Ph.D. Student	2.16	11.20	.19	.85	1.00
Research Assistant-Postdoctoral Researcher	10.32	13.83	.75	.46	1.00
Research Assistant-Administrative and Technical Stuff	-14.15	12.46	-1.14	.26	1.00
Research Assistant- Auxiliary Assistant	-27.19	11.34	-2.40	.02	.35
Ph.D. Student-Postdoctoral Researcher	8.16	13.08	.62	.53	1.00
Ph.D. Student-Administrative and Technical Stuff	-11.99	11.62	-1.03	.30	1.00

Ph.D. Student- Auxiliary Assistant	-25.03	10.41	-2.40	.02	.34
Postdoctoral Researcher- Administrative and Technical Stuff	-3.83	14.17	-.27	.79	1.00
Postdoctoral Researcher- Auxiliary Assistant	-16.86	13.20	-1.28	.20	1.00
Administrative and Technical Stuff- Auxiliary Assistant	-13.03	11.75	-1.11	.27	1.00

Each row tests the null hypothesis that the distributions in sample 1 and sample 2 are equal. Asymptotic significances (2-sided tests) are displayed. The significance level is .05.

a. Significance values are adjusted by the Bonferroni correction for multiple tests.

Pairwise Comparisons of Function – Scale Workplace Insecurity

Sample 1-Sample 2	Test statistic	SE	Standard test statistic	Sig.	Ad. sig.[a]
Professor-Lecturer	-6.75	17.41	-.39	.70	1.00
Professor-Administrative and Technical Stuff	-36.40	15.75	-2.31	.02	.44
Professor- Auxiliary Assistant	-43.56	14.94	-2.92	<.001	.07
Professor-Ph.D. Student	-45.04	14.81	-3.04	<.001	.05
Professor-Research Assistant	-59.45	15.53	-3.83	<.001	.00
Professor-Postdoctoral Researcher	-68.87	17.14	-4.02	<.001	.00
Lecturer-Administrative and Technical Stuff	-29.65	15.42	-1.92	.05	1.00
Lecturer- Auxiliary Assistant	-36.82	14.59	-2.52	.01	.24
Lecturer-Ph.D. Student	-38.30	14.46	-2.65	.01	.17
Lecturer-Research Assistant	-52.70	15.19	-3.47	<.001	.01
Lecturer-Postdoctoral Researcher	-62.12	16.84	-3.69	<.001	.00
Administrative and Technical Stuff-Auxiliary Assistant	-7.16	12.57	-.57	.57	1.00
Administrative and Technical Stuff-Ph.D. Student	8.64	12.41	.70	.49	1.00
Administrative and Technical Stuff-Research Assistant	23.04	13.26	1.74	.08	1.00
Administrative and Technical Stuff-Postdoctoral Researcher	32.47	15.12	2.15	.03	.67
Auxiliary Assistant-Ph.D. Student	1.48	11.37	.13	.90	1.00
Auxiliary Assistant-Research Assistant	15.88	12.28	1.29	.20	1.00

Auxiliary Assistant-Postdoctoral Researcher	25.31	14.27	1.77	.08	1.00
Ph.D. Student-Research Assistant	-14.40	12.13	-1.19	.24	1.00
Ph.D. Student-Postdoctoral Researcher	23.83	14.14	1.69	.09	1.00
Research Assistant-Postdoctoral Researcher	9.43	14.88	.63	.53	1.00

Each row tests the null hypothesis that the distributions in sample 1 and sample 2 are equal. Asymptotic significances (2-sided tests) are displayed. The significance level is .05.

a. Significance values are adjusted by the Bonferroni correction for multiple tests.

Pairwise Comparisons of Function – Number of Sedentary Hours per Day

Sample 1-Sample 2	Test statistic	SE	Standard test statistic	Sig.	Ad. sig.[a]
Lecturer-Administrative and Technical Stuff	-13.74	15.50	-.89	.38	1.00
Lecturer-Research Assistant	-16.44	15.26	-1.08	.28	1.00
Lecturer- Auxiliary Assistant	-18.59	14.63	-1.27	.20	1.00
Lecturer-Professor	20.33	17.26	1.18	.24	1.00
Lecturer-Ph.D. Student	-37.48	14.37	-2.61	.01	.19
Lecturer-Postdoctoral Researcher	-49.00	16.98	-2.89	<.001	.08
Administrative and Technical Stuff-Research Assistant	2.70	13.59	.20	.84	1.00
Administrative and Technical Stuff-Auxiliary Assistant	-4.85	12.88	-.38	.71	1.00
Administrative and Technical Stuff-Professor	6.59	15.81	.42	.68	1.00
Administrative and Technical Stuff-Ph.D. Student	23.74	12.59	1.89	.06	1.00
Administrative and Technical Stuff-Postdoctoral Researcher	35.26	15.50	2.27	.02	.48
Research Assistant- Auxiliary Assistant	-2.16	12.59	-.17	.86	1.00
Research Assistant-Professor	3.90	15.57	.25	.80	1.00
Research Assistant-Ph.D. Student	21.04	12.29	1.71	.09	1.00
Research Assistant-Postdoctoral Researcher	32.56	15.26	2.13	.03	.69
Auxiliary Assistant-Professor	1.74	14.96	.12	.91	1.00

Auxiliary Assistant-Ph.D. Student	18.89	11.50	1.64	.10	1.00
Auxiliary Assistant-Postdoctoral Researcher	30.41	14.63	2.08	.04	.79
Professor-Ph.D. Student	-17.15	14.70	-1.17	.24	1.00
Professor-Postdoctoral Researcher	-28.67	17.26	-1.66	.10	1.00
Ph.D. Student-Postdoctoral Researcher	11.52	14.37	.80	.42	1.00

Each row tests the null hypothesis that the distributions in sample 1 and sample 2 are equal. Asymptotic significances (2-sided tests) are displayed. The significance level is .05.

a. Significance values are adjusted by the Bonferroni correction for multiple tests.

Pairwise Comparisons of Function – Scale of Perceived Stress Symptoms

Sample 1-Sample 2	Test statistic	SE	Standard test statistic	Sig.	Ad. sig.[a]
Professor-Lecturer	-15.00	17.34	-.87	.39	1.00
Professor-Administrative and Technical Stuff	-24.82	15.88	-1.56	.12	1.00
Professor-Research Assistant	-29.32	15.64	-1.87	.06	1.00
Professor-Postdoctoral Researcher	-33.13	17.34	-1.91	.06	1.00
Professor-Ph.D. Student	-41.05	14.71	-2.79	.01	.11
Professor- Auxiliary Assistant	-46.40	15.02	-3.09	<.001	.04
Lecturer-Administrative and Technical Stuff	-9.81	15.57	-.63	.53	1.00
Lecturer-Research Assistant	-14.31	15.33	-.93	.35	1.00
Lecturer-Postdoctoral Researcher	-18.12	17.06	-1.06	.29	1.00
Lecturer-Ph.D. Student	-26.05	14.38	-1.81	.07	1.00
Lecturer- Auxiliary Assistant	-31.40	14.70	-2.14	.03	.69
Administrative and Technical Stuff-Research Assistant	4.50	13.66	.33	.74	1.00
Administrative and Technical Stuff-Postdoctoral Researcher	8.31	15.57	.53	.59	1.00
Administrative and Technical Stuff-Ph.D. Student	16.24	12.58	1.29	.20	1.00
Administrative and Technical Stuff-Auxiliary Assistant	-21.58	12.94	-1.67	.10	1.00
Research Assistant-Postdoctoral Researcher	3.81	15.33	.25	.80	1.00
Research Assistant-Ph.D. Student	11.74	12.28	.96	.34	1.00

Research Assistant- Auxiliary Assistant	-17.08	12.65	-1.35	.18	1.00
Postdoctoral Researcher-Ph.D. Student	-7.92	14.38	-.55	.58	1.00
Postdoctoral Researcher- Auxiliary Assistant	-13.27	14.70	-.90	.37	1.00
Ph.D. Student- Auxiliary Assistant	-5.35	11.48	-.47	.64	1.00

Each row tests the null hypothesis that the distributions in sample 1 and sample 2 are equal. Asymptotic significances (2-sided tests) are displayed. The significance level is .05.

a. Significance values are adjusted by the Bonferroni correction for multiple tests.

TableS7: Identified strengths and potentials from the expert interviews (Gesunde UniBE, Switzerland, 2021)

Block	Themes (n ≥ 3 mentioned)	Sample Quotes
I: Knowledge of health promotion		
	• University Sports (12)	«University Sports is a giant offering that we provide, with virtually all the sports and opportunities that we basically know about.»
	• Healthy diet (5)	«...but also the support of the institution when it comes to healthy food...»
	• Employee health prevention (5)	«To want to maintain health and to put the benefits of a healthy workforce or even students afterwards at the service of the university.»
	• Ergonomic workplace (5)	«Foremost, I think it's good how much attention is paid to ergonomic aspects here.»
	• Occupational safety (4)	«Known in particular are KOPAS (occupational safety contact persons); workplace safety; university sports; and various continuing education programs in this area.»
	• Workload and working time (3)	«...about adequate working hours. You could even take the workload into it...»
	• Working atmosphere (3)	«Actually, the work climate first. It's a healthy work climate. That is, a stress-free work climate, because we have a very firm focus on that from an equality standpoint.»
II: Potential for improvement		
	• Stress prevention and mental health prevention and promotion (12)	«In my environment, I see potential for improvement especially in the area of mental health (i.e. company culture, stress prevention), while I think that in the area of physical activities, nutrition, there are very many offers available.»
	• Reduction of workload and promotion of work-life balance (8)	«An eternal topic is the work-life balance. When I look at my career at the University of Bern, I see enormous progress that has been made there, but it's not yet at the point where it should actually be necessary so that you have really satisfied employees who don't live under stress.»

	• Promotion of a good, health-promoting work culture and leadership culture as well as training-/education of managers on health-promoting leadership (4)	«It is a task of the bosses, the supervisors to care for the employees in their area, that there is a good working atmosphere, that the framework conditions are optimal, that they can develop, that it is a good working atmosphere.»
	• Promotion of physical activities (5)<br>• Promotion of healthy nutrition (3)	«...that you promote healthy eating, that you promote exercise and physical activity, that you also remind people that mental activity is also vital.»
III: Factors for long-term success		
	• Create an attractive and diverse employee health promotion program that increases employee satis-faction and work-life balance (6)	«The goal for me would be to have employees that I can use as long and extensively as possible for my faculty. So I need to make sure that my staff can work in an environment where they are not taken advantage of, sick, or stressed.»
	• Sustainable development of a health policy/health and management culture inclusive training/sensitization of managers for the topic «health of employees at work» (6)	«That, for example, the people who lead institutes or departments are prepared and trained in their personnel tasks, because institute directors in particular are key people, they are the personnel managers in their area.»
	• Promoting employees' awareness of their own health e.g. through counselling/training concepts (4)	«Employees/students must recognize the benefits of the measures that they will be adhered to over the long term.»
	• Integration of sport and exercise into everyday life (3)	«Bringing the movement into focus.»
IV: possible orga-nizational structure		
	• Emergence of a central specialist office or coordination office for health promotion (13)	«Offers can possibly be better networked, informed and communicated by a coordination office.»
V: The most important focus areas regarding health promotion		

for the next few years		
	• Promoting physical activity among employees (7)	«A balance between the relatively seat-heavy work.»
	• Reduction of workload to a tolerable level and promotion of work-life balance (5)	«I think the point of how do we get the workload to a tolerable level- I think that would have to be a strong focus.»
	• Stress management (5)	«At the level of doctoral students and post-docs, we're talking mainly about stress. And how do I deal with performance, with performance demands? How can I envision myself in a career situation? And how do I get support? How do I become resilient to situations where I have to constantly ask myself, «Am I actually doing the right thing now, or am I doing the wrong thing?»
	• Healthy diet (5)	«We are trying to make healthy eating a topic for cafeterias.»
	• Improving the job satisfaction of employees (3)	«I believe in the topic of employee satisfaction. Also trying to get a work-life balance, especially with the mid-level staff.»
VI: Possible benefits, wishes and demands		
	• Better image of the university of Berne (3).	«So such a very great state of affairs, of course, would be if the University of Bern had a reputation for managing and implementing this issue in a sensible and smart way.»
VII: Collaboration opportunities	All interview participants see collaboration opportunities between the «Healthy University of Bern» initiative and its offices/departments, as well as the faculties at the University of Bern.	No quotes due to confidentiality (Nc.)

Table S8:Answers to strengths and potentials from the expert interviews in German (Gesunde UniBE, Switzerland, 2021)

Block	Themes (n ≥ 3 mentioned)	Sample Quotes
I: Knowledge of health promotion		
	• University Sports (12)	«Der Uni-Sport ist ein riesen Angebot, das wir bieten, mit praktisch allen Sportarten und Möglichkeiten, die wir im Grunde genommen kennen.»
	• Healty diet (5)	«…aber auch die Unterstützung der Institution, wenn es um gesundes Essen geht…»
	• Employee health prevention (5)	«Gesundheit erhalten zu wollen und die Vorteile einer gesunden Mitarbeiterschaft oder auch Studierendenschaft nachher in den Dienst der Universität zu stellen.»
	• Ergonomic workplace (5)	«Das ich erstmal gut finde, wie stark hier auf ergonomische Gesichtspunkte geachtet wird.»
	• Occupational safety (4)	«Bekannt sind insbesondere KOPAS (Kontaktpersonen der Arbeitssicherheit); Arbeitsplatzsicherheit; Uni-Sport sowie verschiedene Weiterbildungsprogramme in diesem Bereich.»
	• Workload and working time (3)	«…über adäquate Arbeitszeiten. Man könnte sogar die Arbeitsbelastung hineinnehmen…»
	• Working atmosphere (3)	«Eigentlich das Arbeitsklima zuerst. Es ist ein gesundes Arbeitsklima. Das heißt ein stressfreies Arbeitsklima, weil wir haben sehr fest von der Gleichstellung her den Fokus darauf.»
II: Potential for improvement		
	• Stress prevention and mental health prevention and promotion (12)	«In meinem Umfeld sehe Verbesserungspotenzial insb. im Bereich der psychischen Gesundheit (d.h. Betriebskultur, Stressprävention), während ich denke, dass im Bereich körperliche Aktivitäten, Ernährung sehr viele Angebote zur Verfügung stehen.»
	• Reduction of workload and promotion of work-life balance (8)	«Ein ewiges Thema die Work-Life-Balance. Wenn ich meine Karriere unter der Universität Bern anschaue, dann sehe ich enorme Fortschritte, die dort gemacht wurden, aber es ist noch nicht an dem Punkt, wo es eigentlich notwendig sein müsste, damit man wirklich zufriedene Mitarbeiter hat, die nicht unter Stress leben.»
	• Promotion of a good, health-promoting work culture and leadership culture as well as training/education of managers on health-promoting leadership (4)	«Es ist eine Aufgabe der Chefs, der Vorgesetzten fürsorglich zu sein für die Mitarbeitenden in ihrem Bereich, dass ein gutes Arbeitsklima herrscht, dass die Rahmenbedingungen optimal sind, dass die sich entfalten können, dass es ein gutes Arbeitsklima ist.»
	• Promotion of physical activities (5)<br>• Promotion of healthy nutrition (3)	«…dass man gesundes Essen fördert, dass man Sport und körperliche Aktivität fördert, dass man auch die Leute daran erinnert, dass auch die mentale Aktivität sehr wichtig ist.»
III: Factors for long-term sucess		
	• Create an attractive and diverse employee health promotion program that increases employee satisfaction and work-life balance (6)	«Das Ziel für mich wäre, dass ich Mitarbeiter habe, die ich möglichst lange und ausgiebig für meine Fakultät brauchen kann. Also muss ich sicherstellen, dass meine Mitarbeiter in einem Umfeld arbeiten können, wo sie nicht ausgenutzt, krank oder gestresst sind.»
	• sustainable development of a health policy/health and management culture incl. training/sensitization of	«Das man zum Beispiel die Personen, die Institute oder Departemente führen in ihrer Personalaufgabe darauf vorbereitet und schult, weil gerade Institutsleiter und

	managers for the topic «health of employees at work» (6)	Leiterinnen sind Schlüsselpersonen, sie sind die Personalverantwortlichen in ihrem Bereich.»
	• Promoting employees' awareness of their own health (4) e.g. through counseling/training concepts	«Die Mitarbeitenden/Studenten müssen den Nutzen der Massnahmen erkennen, dass sie auch langfristig eingehalten werden.»
	• Integration of sport and exercise into everyday life (3)	«Die Bewegung in den Fokus nehmen.»
IV: possible organizational structure		
	• Emergence of a central specialist office or coordination office for health promotion (13)	«Angebote können ev. durch eine Koordinationsstelle besser vernetzt, informiert und kommuniziert werden.»
V: The most important focus areas regarding health promotion for the next few years		
	• Promoting physical activity among employees (7)	«Ein Ausgleich zwischen dem relativ sitzlastigen Arbeiten.»
	• Reduction of workload to a tolerable level and promotion of work-life balance (5)	«Ich glaube den Punkt, wie schaffen wir es, dass wir die Arbeitsbelastung auf ein erträgliches Maß bringen- ich glaube das müsste ein starker Fokus sein.»
	• Stress management (5)	«Auf der Ebene Doktoranden und Post-Docs, reden wir vor allem von Stress. Und wie gehe ich mit Leistung, mit Leistungsanforderungen um? Wie kann ich mich in eine Karriere Situationen hineindenken? Und wie kriege ich Unterstützung? Wie werde ich Resilienz gegenüber Situationen, wo ich mir dauernd die Frage stellen muss: "Mach ich jetzt eigentlich das Richtige oder mache ich das Falsche?»
	• Healthy diet (5)	«Wir versuchen, gesunde Ernährung zu einem Thema in den Kantinen zu machen.»
	• Improving the job satisfaction of employees (3)	«Ich glaube auf die Mitarbeiterzufriedenheit. Auch den Versuch, eine Work-Life-Balance hinzubekommen, insbesondere beim Mittelbau.»
VI: Possible benefits, wishes and demands		
	• Better image of the university of berne (3).	«Also so ein ganz toller Zustand wäre natürlich, wenn die Universität Bern den Ruf hätte, auf eine vernünftige und clevere Weise dieses Thema zu bewirtschaften und umzusetzen.»
VII: Collaboration opportunities	All interview participants see collaboration opportunities between the «Healthy University of Bern» initiative and its offices/departments, as well as the faculties at the University of Bern.	No quotes due to confidentiality

Table S9: Potentials of the Reflection and Evaluation Instrument areas at the University of Bern (Gesunde UniBE, Switzerland, 2021)

Potentials: organizational structures and processes	Effort	Benefit
Integrating health promotion in the mission statement and strategy of the University of Bern	Middle	Middle – High
Active engagement of the university leads	Middle	High
Provision of financial and personnel resources	Middle – High	High
Increased integration of health promotion into university structures.	Middle	Middle – High
Gradual expansion as well as integration of health promotion in service processes, teaching, and research.	High	Middle
Expansion of the steering committee and inclusion of other departments after successful pilot phase at the Faculty of Human Sciences	Low – Middle	Middle – High
Develop a wholistic definition of health	Middle	High
**Potentials: existing analyses**		
Combination or alternating implementation of the questionnaire created by the needs analysis and the existing employee survey by Empiricon at the entire University of Bern	Low – Middle	Middle – High
Establishment of the created survey instrument by the Network of Health Promoting Universities in Switzerland	Low	Middle – High
**Potentials: internal and external communication**		
Internal and external communication of the cross-cutting topic of «health» through a key visual that is integrated into the corporate design of the University of Bern	Low – Middle	Middle – High
Establishment of action days at the University of Bern	Middle	Middle
Raising awareness among status groups regarding health promotion and promotion of existing and new health-promoting offerings	Low – Middle	Middle – High
Collaborate with the steering group in planning and implementing strategic and sustainable motivational «nudges» for healthy choices at decision points such as elevators	Low – Middle	Middle – High
Communication of results from analyses and activities at network meetings and workshops	Low – Middle	Middle
**Potentials: health promotion and prevention measures**		
Enabling access to on-site counselling	Low – Middle	Low – Middle
Intensified promotion of the counselling services offered by the Counselling Center of the Bernese Universities.	Low – Middle	Middle – High

Expansion of offerings for employees through collaboration with clinical psychology	Middle – High	Middle
Regular exchange between the steering committee and the consulting centres.	Low	Middle
Expansion of university sports offerings to include exercise promotion and decentralized offerings.	Middle	Middle – High
Expanding behavioural offerings (e.g., healthy eating, stress management, sedentary behaviour).	Middle – High	High

Table S10: Analyzed documents with policies that relate directly or indirectly to the promotion and prevention of university employee health and well-being (Gesunde UniBE, Switzerland, 2021).

	Number of Documents	*Number of Policies*
**Total**	36	55
**Direct relation**	9	9
• Promotion of physical and mental health	4	4
• Promotion of well-being	1	1
• Promotion or sustainability of social resources	4	4
**Indirect relation**	24	46
• Promotion of good working conditions	5	6
• Dealing with conflicts	1	1
• Promotion of leadership skills and a trusting and appreciative leadership culture	4	4
• Promoting compatibility of family and career	9	9
• Promotion of equality	8	9
• Promotion of further education and development	3	4
• Promotion of young academics	8	9
• Promotion of exercise and sports	2	4
• Promotion of healthy nutrition	1	1

Table S11: Score of the 12 sites regarding health promoting infrastructures (Gesunde UniBE, Switzerland, 2021).

**Site**	Physical activity, exercise, and sports [0 - 18]	Nutrition [0 - 27.5]	Relaxation and Recreation [0 - 13]	Smoking and secondhand smoke [0 - 6]	Information Environment [0 - 12]
Area 1	8.7	14.5	7.0	0	0
Area 2	10.4	*	11.5	0	0
Area 3	6.5	10.8	7.0	0	2.0
Area 4	5.3	11.0	6.5	0	3.0
Area 5	10.4	*	7.5	0	0
Area 6†	2.8	*	6.5	0	0
Area 7	10.5	15.4	11.5	0	1.0
Area 8	7.8	*	10.5	0	2.0
Area 9	8.1	11.0	10.5	0	2.0
Area 10	10.1	12.0	12.0	4.0	6.0
Area 11	13.6	12.7	12.0	0	1.0
Area 12	13.6	14.8	12.0	5.5	2.0

* Locations without cafeteria or refectory = no score calculated
† Renovation work was taking place at the time of the survey.

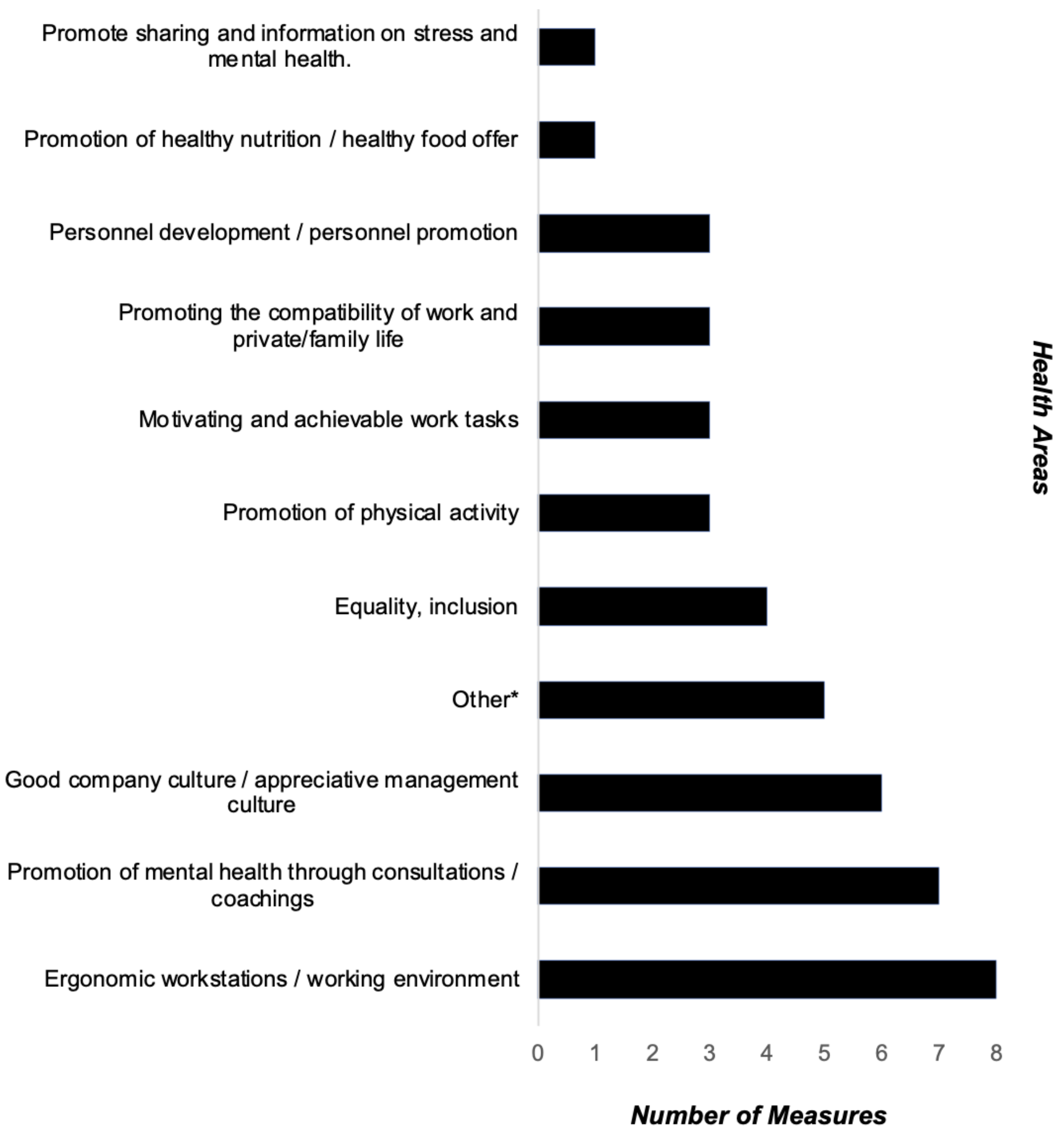

*advice on addiction; skin and hand protection; occupational health and safety; conditions of employment; information on workplace health promotion

Figure S1: Number of Measures per Health Area (GesundeUniBE, Switzerland, 2021)

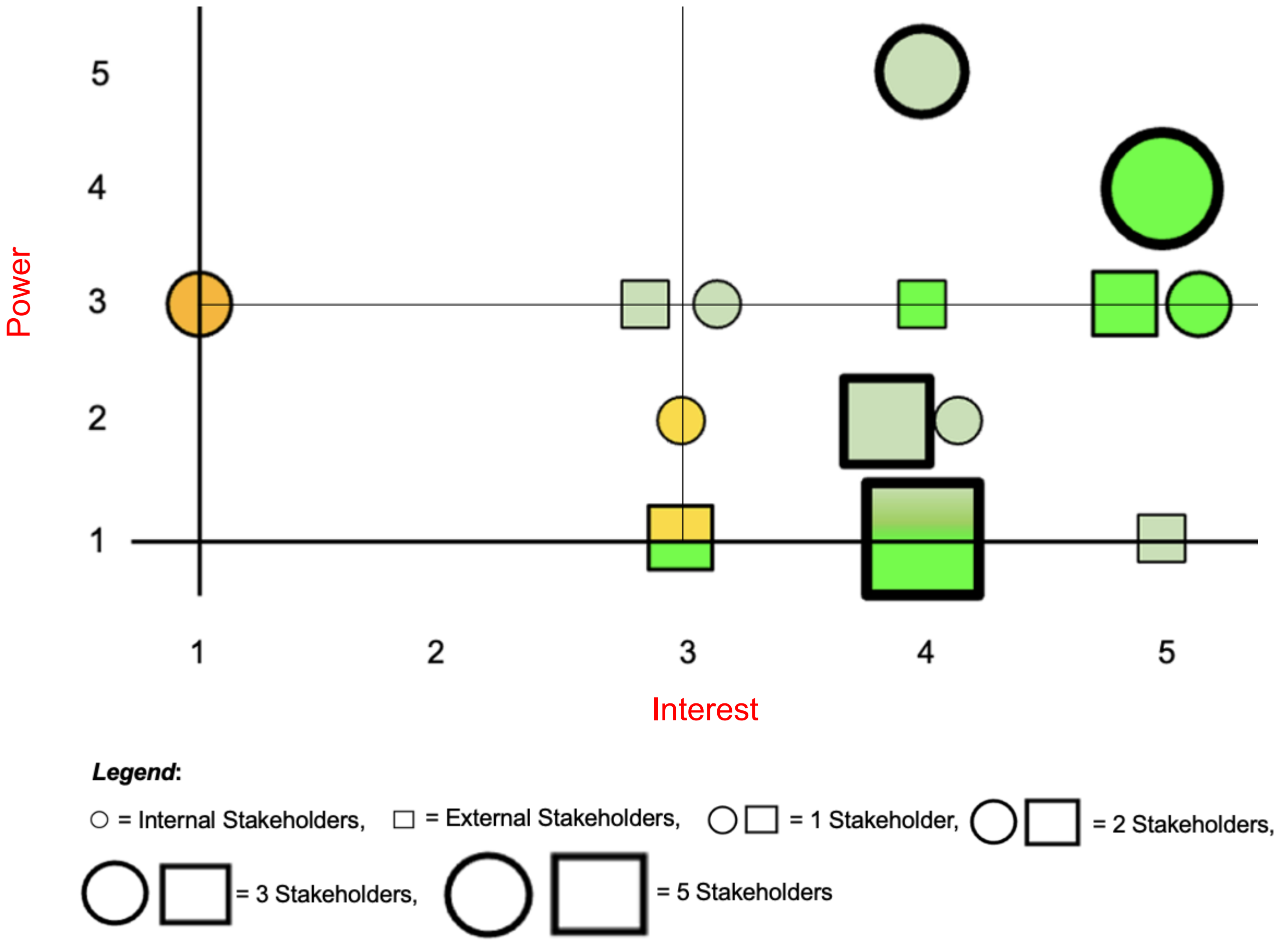

Attitude towards Initiative				
negative	Slightly negative	neutral	Slightly positive	positive

Figure S2: Categorized Internal and External Stakeholders (GesundeUniBE, Switzerland, 2021)
